# Predicting Anatomical Therapeutic Chemical (ATC) Classification of Drugs by Integrating Chemical-Chemical Interactions and Similarities

**DOI:** 10.1371/journal.pone.0035254

**Published:** 2012-04-13

**Authors:** Lei Chen, Wei-Ming Zeng, Yu-Dong Cai, Kai-Yan Feng, Kuo-Chen Chou

**Affiliations:** 1 College of Information Engineering, Shanghai Maritime University, Shanghai, China; 2 Institute of Systems Biology, Shanghai University, Shanghai, China; 3 Shanghai Center for Bioinformation Technology, Shanghai, China; 4 Key Laboratory of Systems Biology, Shanghai Institutes for Biological Sciences, Chinese Academy of Sciences, Shanghai, China; 5 Gordon Life Science Institute, San Diego, California, United States of America; Koç University, Turkey

## Abstract

The Anatomical Therapeutic Chemical (ATC) classification system, recommended by the World Health Organization, categories drugs into different classes according to their therapeutic and chemical characteristics. For a set of query compounds, how can we identify which ATC-class (or classes) they belong to? It is an important and challenging problem because the information thus obtained would be quite useful for drug development and utilization. By hybridizing the informations of chemical-chemical interactions and chemical-chemical similarities, a novel method was developed for such purpose. It was observed by the jackknife test on a benchmark dataset of 3,883 drug compounds that the overall success rate achieved by the prediction method was about 73% in identifying the drugs among the following 14 main ATC-classes: (1) alimentary tract and metabolism; (2) blood and blood forming organs; (3) cardiovascular system; (4) dermatologicals; (5) genitourinary system and sex hormones; (6) systemic hormonal preparations, excluding sex hormones and insulins; (7) anti-infectives for systemic use; (8) antineoplastic and immunomodulating agents; (9) musculoskeletal system; (10) nervous system; (11) antiparasitic products, insecticides and repellents; (12) respiratory system; (13) sensory organs; (14) various. Such a success rate is substantially higher than 7% by the random guess. It has not escaped our notice that the current method can be straightforwardly extended to identify the drugs for their 2^nd^-level, 3^rd^-level, 4^th^-level, and 5^th^-level ATC-classifications once the statistically significant benchmark data are available for these lower levels.

## Introduction

Nowadays, the **A**natomical **T**herapeutic **C**hemical (ATC) classification system, recommended by the World Health Organization (WHO), is the most widely recognized classification system for drugs. This classification system divides drugs into different groups according to the organ or system on which they act and/or their therapeutic and chemical characteristics. Accordingly, the ATC classification is very helpful for studying utilization of drugs and categorizing them according to different purposes, therapeutic properties, chemical and pharmacological properties (see Report of the WHO Expert Committee, 2005; World Health Organ Tech Rep, Ser:1–119). In the ATC classification system, drugs are classified into 14 main classes (http://www.whocc.no/atc/structure_and_principles/). In order to understand this kind of complicated classification system, some efforts have been made [1], [2]. In a pioneer study, Gurulingappa et al. [2] proposed a method to study the ATC-classification system by combining the information extraction and machine learning techniques. However, their method can be used to identify the drug compounds only within the class of “Cardiovascular System”, one of the 14 main ATC classes.

During the past decade, many compound databases, such as KEGG (Kyoto Encyclopedia of Genes and Genomes) [3], [4], have been established. From these databases many compounds and their properties can be acquired. Such abundant informations provide an opportunity to analyze ATC classification system in greater detail. Encouraged by the successes of using machine learning and data mining methods to investigate complicated problems in a variety of biological areas [5], [6], [7], [8], [9], the present study was initiated in an attempt to develop a powerful method by which one can identify query drugs compound among all their 14 posible main classes.

According to a recent comprehensive review [10], to establish a really useful statistical predictor for a biological system, we need to consider the following procedures: (i) construct or select a valid benchmark dataset to train and test the predictor; (ii) formulate the samples concerned with an effective mathematical expression that can truly reflect their intrinsic correlation with the target to be predicted; (iii) introduce or develop a powerful algorithm (or engine) to operate the prediction; (iv) properly perform cross-validation tests to objectively evaluate the anticipated accuracy of the predictor. Below, let us describe how to deal with these steps one by one.

## Materials and Methods

Recently, the information of protein-protein interactions have been used for predicting various attributes of proteins (see, e.g., [11], [12], [13]), implying that interactive proteins are more likely to share common biological functions [11] than non-interactive ones [14]. Likewise, it is more likely that two interactive drug compounds may have the similar biological function. Actually, it is generally accepted that compounds with similar physicochemical properties often involve in similar biological activities [1]. Accordingly, it is reasonable to assume that the interactive drugs may likely belong to the same ATC-class, and so do those drugs with similar structures. Based on such rational, let us construct the following benchmark to develop a new method for identifying the ATC-classes of drugs.

### Benchmark Dataset

The dataset for drugs was obtained from the public available database KEGG [3], [4] at ftp://ftp.genome.jp/pub/kegg/medicus/drug/drug (June, 2011). There are totally 9,758 drugs. After excluding those without the information of ATC-codes, the remaining are 4,376 drug samples, from which further screening was performed to remove those without the information of both chemical-chemical interactions and chemical-chemical similarities. After the above winnowing procedures, we finally obtained the benchmark dataset 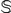 containing 3,883 drugs classified into 14 main ATC-classes, as can be formulated by
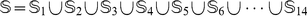
(1)where 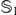 represents the subset for the 1^st^ main ATC class called “Alimentary tract and metabolism”, 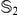 the subset for the 2^nd^ main ATC class “Blood and blood forming organs”, 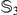 the subset for the 3^rd^ main ATC class “Cardiovascular system”, and so forth (cf. **Table 1**); while 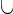 represents the symbol for “union” in the set theory. For convenience, hereafter let us just use *C*_1_, *C*_2_, *C*_3_, …, *C*_14_ as the tags of the 14 classes. A breakdown of the 3,883 drugs into the 14 main ATC-classes is given in **Table 1**. For the codes of these drugs in each of the 14 classes, see Supporting Information S1. During the course of constructing the benchmark dataset, the information from http://www.genome.jp/kegg-bin/get_htext?br08303.keg was used that collected the drug compounds and their ATC classification information from http://www.whocc.no/atc_ddd_index/ and provided the ATC code for each drug.

**Table 1:** Breakdown of the benchmark dataset 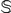 according to the 14 main ATC classes.

Tag	The 1^st^-level ATC class	Number of drugs
*C*_1_	Alimentary tract and metabolism	540
*C*_2_	Blood and blood forming organs	133
*C*_3_	Cardiovascular system	591
*C*_4_	Dermatologicals	421
*C*_5_	Genito-urinary system and sex hormones	248
*C*_6_	Systemic hormonal preparations, excluding sex hormones and insulins	126
*C*_7_	Antiinfectives for systemic use	521
*C*_8_	Antineoplastic and immunomodulating agents	232
*C*_9_	Musculo-skeletal system	208
*C*_10_	Nervous system	737
*C*_11_	Antiparasitic products, insecticides and repellents	127
*C*_12_	Respiratory system	427
*C*_13_	Sensory organs	390
*C*_14_	Various	211
Number of total virtual drugs 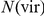		4,912^a^
Number of total structural different drugs 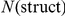		3,883^b^

a See Eqs.2–3 for the definition about the number of virtual drugs, and its relation with the number of structural different drugs.

b Of the 3,883 structural different drugs, 3,295 belong to one class, 370 to two classes, 110 to three classes, 37 to four classes, 27 to five classes, and 44 to six classes. See Supporting Information S1 for the detailed drug codes listed in each of 14 ATC-classes.

Because some drugs may belong to more than one main ATC-class, like the case in dealing with proteins with multiple location sites [15], [16], [17], it is instructive to introduce the concept of the “virtual drugs” as illustrated as follows. A drug compound belonging to two different ATC-classes will be counted as 2 virtual samples even though they have an identical chemical structure; if belonging to three different classes, 3 virtual samples; and so forth. Accordingly, the total number of the different virtual drug samples is generally greater than that of the total different structural drug samples. Their relationship can be formulated as follows [18]

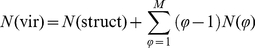
(2)where 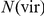 is the number of total different virtual drug samples in 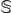, 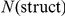 the number of total different structural drugs, 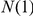 the number of drugs belonging to one ACT-class, 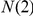 the number of drugs belonging to two ATC-classes, and so forth; while 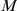 is the number of total main ACT-classes (for the current case, 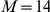 (cf. **Table 1**).

**Figure 1:**
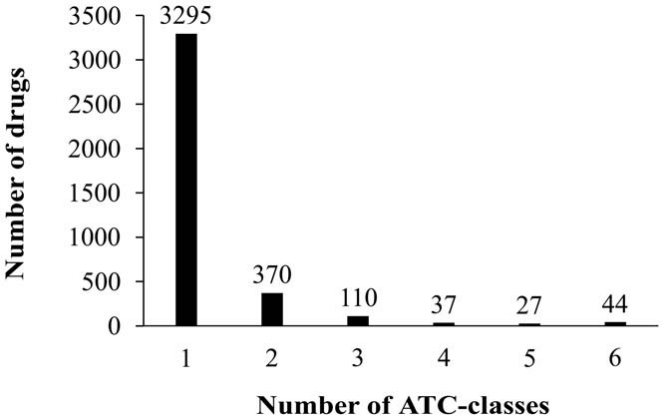
An illustration to show the distribution about the numbers of ATC-classes a same drug may belong to. For the 3,883 drugs in 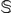, 3,295 belong to one class, 370 to two classes, 110 to three classes, 37 to four classes, 27 to five classes, 44 to six classes, and none to seven or more classes.

For the current 3,883 drugs in 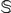, 3,295 occur in one class, 370 in two classes, 110 in three classes, 37 in four classes, 27 in five classes, 44 in six classes, and none in seven or more classes (**Figure 1**). Substituting these data into **Eq.1**, we have
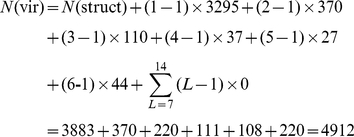
(3)which is fully consistent with the figures in **Table 1** and the data in Supporting Information S1.

### Prediction Based on Chemical-Chemical Interactions

Based on the fact that the interactive compounds often involve in similar biological activities [11], it is feasible to predict the ATC-class of a query drug using the information of chemical-chemical interactions, as described below.

STITCH (Search tool for interactions of chemicals) [19] is a large database containing known and predicted interactions between chemicals and between proteins derived from experiments, literature and other databases. We downloaded the information of chemical-chemical interactions from http://stitch.embl.de:8080/download/chemical_chemical.links.v2.0.tsv.gz. Each of these interactions was evaluated by a confidence score, ranging from 1 to 1000, to reflect the likelihood of its occurrence. For any two drugs *d*_1_ and *d*_2_, their interaction confidence score was denoted by 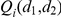. Particularly, if the interaction between *d*_1_ and *d*_2_ does not exist in STITCH, their interaction confidence score was set as zero, *i.e.*, 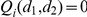.

Suppose that a training dataset 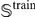 consists of *n* drugs 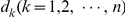, and that the 14 main ATC-classes are denoted by 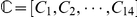, where *C*_1_ represents “Alimentary tract and metabolism”, *C*_2_ “Blood and blood forming organs”, and so forth (see **Table 1**). The ATC-classes of any drug *d_i_* can be formulated as
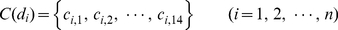
(4)where
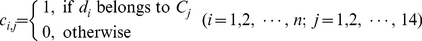
(5)According to the chemical-chemical interaction approach, the likelihood for a query drug 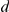 belonging to *C_j_*, denoted as 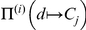, can be calculated by
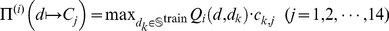
(6)where 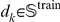 means that 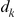 is an element of the training dataset 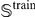. According **Eq.6**, the likelihood that 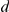 belongs to *C_j_* can be formulated as the maximum of the interaction confidence scores between 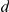 and those drugs that belong to *C_j_* in the training dataset 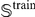. Obviously, the larger the score is, the more likely that 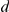 belongs to 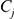. When 
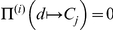
, it means that the probability for the drug 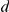 belonging to the class *C_j_* is zero. Given a query drug compound 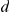, suppose the outcome derived from **Eq.6** is
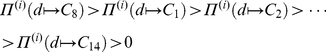
(7)which means that the highest probability for the drug 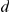 belonging to the ATC-class is 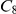 (“Antineoplastic and immunomodulating agents”), followed by 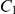 (“Alimentary tract and metabolism”), and so forth (cf. **Table 1**). If there is a tie between two terms in **Eq.7**, then the probabilities for the drug belonging to the two corresponding classes are the same. But this kind of tie case rarely happened.

Note that the outcome of **Eq.6** might turn out to be trivial, i.e.,
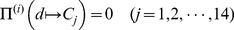
(8)indicating that no chemical-chemical interaction exists for the query drug 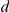 in the training dataset 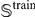; i.e.,
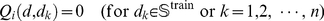
(9)Under such a circumstance, no meaningful result would be obtained by the “interaction-based” method, and we should instead use the “similarity-based method as described in the next section.

### Prediction Based on Chemical-Chemical Similarities

Likewise, based on the fact that the compounds with similar physicochemical properties often have the same biological activities [1], we can also use the information of chemical-chemical similarities as another feasible avenue to predict the ATC-class for a query drug. To realize this, let us first introduce how to use graphical representation to measure the similarity between two drug compounds.

Graphical approaches can provide intuitive pictures and useful insights for studying and analyzing complicated biological systems, as demonstrated by many studies on a series of important biological topics (see, e.g., [20], [21], [22], [23], [24], [25], [26], [27], [28], [29], [30]). Here, a special graphic approach was utilized to estimate the similarity of two compounds. Hattori *et al.* [31] first proposed a means to measure the similarity of two compounds via their graph representations. Since each chemical structure can be easily represented by a 2D (two-dimensional) graph where vertices stand for atoms and edges for bonds between them, the similarity of two compounds can be estimated by the Jaccard coefficient [32], [33] based on their maximum common subgraph. The similarity scores between compounds by this method can be obtained from the website at http://www.genome.jp/ligand-bin/search_compound. According to the graphical method by Hattori *et al.* [31], given two drug compounds *d*_1_ and *d*_2_, their similarity score was denoted by 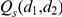. When the similarity score between *d*_1_ and *d*_2_ does not exist in http://www.genome.jp/ligand-bin/search_compound, their similarity was set as zero; *i.e.*, 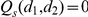.

Thus, the prediction method based on the chemical-chemical similarities can be formulated in a way almost completely parallel to that of the chemical-chemical interactions as done in the preceding section.

Now, instead of Eq.6, we have
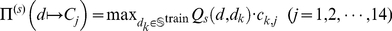
(10)where the superscript and subscript “s” stands for the 1^st^ letter of “similarity”, implying that the calculation is now based on “chemical-chemical similarity” instead of “chemical-chemical interaction” as done in **Eq.6**.

### Prediction by Integrating the Interaction-Based and Similarity-Based Methods

Given a query drug compound 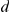, when the integrated method was used to identify its ATC-class, the prediction involved the following two steps.

#### Step 1

The interaction-based method (cf. **Eq.6**) was first applied to identify its ATC-class.

#### Step 2

If the probabilities thus obtained for the drug belonging to all the 14 ATC-classes were zero as indicated in **Eq.8**, meaning no meaningful results were obtained at all, then the prediction would continue using the similarity-based method (cf. **Eq.10**).

### Jackknife Cross-Validation

In statistical prediction, the following three cross-validation methods are often used to examine the quality of a predictor: independent dataset test, subsampling (or k-fold crossover) test, and jackknife test [34]. However, of the three test methods, the jackknife test is deemed the least arbitrary that can always yield a unique result for a given benchmark dataset [35]. The reasons are as follows. (i) For the independent dataset test, although all the samples used to test a predictor are outside the training dataset used to train the prediction engine so as to exclude the “memory” effect or bias, the way of how to select the independent samples for testing the predictor could be quite arbitrary unless the number of independent samples is sufficiently large. This kind of arbitrariness might lead to completely different conclusions. For instance, a predictor achieving a higher success rate than the other for a given independent testing dataset might not able to keep so when tested by another independent testing dataset [34]. (ii) For the subsampling (or k-fold crossover) test, the concrete procedure usually used in literatures was the 5-fold, 7-fold or 10-fold cross-validation. The problem with this kind of subsampling test was that the number of possible selections in dividing a benchmark dataset would be an astronomical figure even for a very simple dataset, as elucidated in [35] and demonstrated by Eqs.28–30 in [10]. Therefore, in any practical subsampling cross-validation tests, only an extremely small fraction of the possible selections were taken into account. Since different selections would always yield different results even for a same benchmark dataset and a same predictor, the subsampling test could not avoid the arbitrariness either. A test method unable to generate a unique outcome should not be deemed as a good one. (iii) In the jackknife test, all the samples in the benchmark dataset will be singled out one-by-one and tested by the predictor trained by the remaining samples. During the process of jackknifing, both the training dataset and testing dataset are actually open, and each sample will be in turn moved between the two. The jackknife test can exclude the “memory” effect. Also, the arbitrariness problem as mentioned above for the independent dataset test and subsampling (or k-fold crossover) test can be avoided because the outcome obtained by the jackknife cross-validation is always unique for a given benchmark dataset. Accordingly, the jackknife test has been widely recognized and increasingly adopted by many investigators to examine the quality of various predictors (see, e.g., [36], [37], [38], [39], [40], [41], [42], [43], [44], [45], [46], [47]). Accordingly, in this study we are to use the jackknife test to examine the prediction quality as well.

### Accuracy Measurement

For any given set of query drugs, we can obtain a series of candidate ATC-classes using the aforementioned prediction methods. Ranked by the likelihood according to their descending order, the prediction accuracy can be defined as
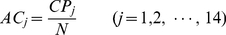
(11)where *CP_j_* denotes the number of drugs whose *j*-th order predicted ATC-class is one of the true ATC-class, and *N* denotes the total number of query drugs whose ATC-classes are to be identified. According to such a definition, the result of higher *AC_j_* with smaller *j* or lower *AC_j_* with larger *j* indicates that the predicted hits are more concentrated meaning a better prediction. Obviously, the result with high 1^st^-order prediction accuracy *AC*_1_ always represents a good quality of prediction.

The average number of ATC-classes for the *N* query drugs is defined as
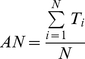
(12)where *T_i_* is the number of ATC-classes for the *i*-th query drug. Thus, another parameter for measuring the proportion of the true classes successfully identified by the first *m*-order prediction hits can be calculated as [13]

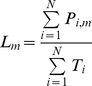
(13)where *P_i,m_* denotes the number of the first *m* predicted candidate ATC-classes that are the true ATC-classes for the *i*-th drug in the dataset. Usually, *m* could take the smallest integer that is equal to or greater than *AN*; *i.e.*,
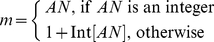
(14)where the operator Int means taking the integer part of the quantity right after it. Again, the result of larger *L_m_* with smaller *m* implies a better prediction with less uncertainty.

## Results and Discussion

For clarity, the original benchmark dataset 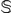 of 3,883 drugs (cf. Supporting Information S1) can be separated into two subsets; i.e.,
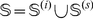
(15)where 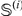 contains 2,144 drugs that had the chemical-chemical interaction information, while 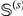 contains 
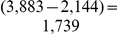
 drugs that had no chemical-chemical interaction information. Listed in **Table 2** are the results obtained by the aforementioned three different prediction methods in identifying the 14 main ATC classes for the drugs investigated. By examining the table, we can observe the following.

**Table 2:** The jackknife success rates by three different methods in identifying the drugs among the 14 main ATC-classes.

Prediction order	Interaction-based^a^	Similarity-based^b^	Integrated^c^
1	67.72%	78.49%	72.55%
2	21.13%	18.86%	20.11%
3	13.43%	8.63%	11.28%
4	7.18%	5.23%	6.31%
5	4.76%	2.88%	3.91%
6	3.54%	1.73%	2.73%
7	1.63%	0.12%	0.95%
8	0.75%	0.35%	0.57%
9	0.75%	0.12%	0.46%
10	0.56%	0.06%	0.33%
11	0.09%	0.00%	0.05%
12	0.28%	0.00%	0.15%
13	0.09%	0.00%	0.05%
14	0.05%	0.00%	0.03%

a Using **Eq.6** on the 2,144 drugs in the benchmark dataset 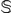 that had the chemical-chemical interaction information.

b Using **Eq.10** on the 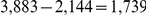 drugs in the benchmark dataset 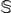 that had no chemical-chemical interaction information.

c Using the integrated method by hybridizing **Eq.6** and **Eq.10** on the 3,883 drugs in the benchmark dataset 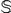 as given in Supporting Information S1.

### Performance of the Interaction-Based Method

For the 2,144 drugs in 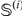 we could use **Eq.6** to conduct the prediction. The results thus obtained are listed in column 2 of **Table 2**, from which we can see that the 1st-order prediction by the jackknife test on the 2,114 drugs was 67.72%. The success rates generally followed a descending trend with increasing of the order number, indicating that the predicted ATC-classes were well sorted for each of the samples investigated. The average number of the ATC-classes in 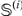 was 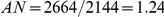 (see **Eq.12**). Thus, it follows according to **Eq.14** that 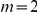, meaning that the first 2-order predictions should be taken into consideration. Substituting these data into **Eq.13**, we obtained the overall success rate by the predictions of the first two orders for the 2,144 drugs in 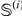 was 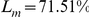, indicating that the interaction-based method is quite promising in identifying the ATC-classed of drugs. However, this method could only be used to deal with those drugs that had the chemical-chemical interaction information.

### Performance of Similarity-Based Method

For the remaining 1,739 drugs in the dataset 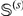 (cf. **Eq.15**) that did not have the chemical-chemical information, the similarity-based method (cf. **Eq.10**) was used as a backup, and the results thus obtained are shown in column 3 of **Table 2**. It can be seen from there that the 1^st^-order prediction by the jackknife test on the 1,739 drugs was 78.49%. The average number of ATC-classes for the drugs in 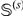 was 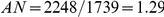 (see **Eq.12**), and hence we have 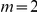 (**Eq.14**), meaning that the first 2-order predictions should be taken into account. Substituting these data into **Eq.13**, we obtained the overall success rate by the first two orders predictions for the 1,739 drugs without the chemical-chemical interaction information was 75.31%, indicating that the similarity-based method was quite promising as well.

At a first glance at **Table 2**, it looks like that the success rates by the similarity-based method (**Eq.10**) are higher than those by the interaction-based method (**Eq.6**). However, since the success rates by the two methods as reported in **Table 2** were derived from two different datasets, 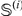 and 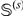 (cf. **Eq.15**) respectively, they might not able to reflect the true superiority between the two methods. To make a comparison between them in a more fair manner, let us construct a new dataset, denoted as 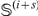. It consists of 2,138 drugs with each containing both chemical-chemical interaction and chemical-chemical similarity informations. The details of such a dataset is given in Supporting Information S2.

Listed in **Table 3** are the results obtained by the methods in identifying the 14 main ATC classes for the 2,138 drugs in the 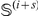
dataset. As we can see from the table, the 1^st^-order prediction accuracy by the interaction-based method was 67.40%, while that by the similarity-based method was 40.36%.

**Table 3:** A comparison between the similarity-based method (Eq.10) and the interaction-based method (Eq.6) in identifying the 2,138 drugs in the 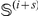 dataset (cf. Supporting Information S2).

Prediction order	Similarity-based	Interaction-based	Difference
1	40.36%	67.40%	27.04%
2	13.89%	21.09%	7.20%
3	9.17%	13.47%	4.30%
4	5.99%	7.16%	1.17%
5	3.32%	4.91%	1.59%
6	2.76%	3.46%	0.70%
7	0.65%	1.54%	0.89%
8	0.23%	0.75%	0.52%
9	0.09%	0.75%	0.66%
10	0.05%	0.56%	0.51%
11	0.05%	0.09%	0.04%
12	0.00%	0.33%	0.33%
13	0.09%	0.09%	0.00%
14	0.05%	0.05%	0.05%

The average number of ATC-classes for the drugs in 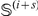 was 1.24 (see **Eq.12**), and hence we have 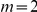 (**Eq.14**), meaning that the first 2-order predictions should be taken into account. Substituting these data into **Eq.13**, we obtained the overall success rate by the 1^st^ two orders predictions for the 2,138 drugs in 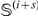 by the interaction-based method (**Eq.6**) was 71.26%, while that by the similarity-based method (**Eq.10**) was only 43.69%, indicating that the interaction-based method is superior to the similarity-based method in identifying the ATC-classes of drugs. That is why in the integrated method the first step was to use the interaction method (**Eq.6**) to identify the ATC-classes for any query drugs. When, and only when no meaningful result was obtained by the interaction-based method, was the similarity-based method (**Eq.10**) used as a backup to continue the prediction (see the Section of “Prediction by Integrating the Interaction-Based and Similarity-Based Methods”).

### Performance of Integrated Prediction Method

Shown in the 4^th^ column of **Table 2** are the results obtained by the integrated method in identifying the 14 main ATC classes for the 3,883 drugs in the benchmark dataset 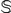. As we can see there, the 1^st^-order prediction accuracy was 72.55%. The average numbers of ATC-classes for the drugs in 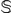 was 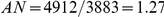 (see **Eq.12**). Thus, it follows according to **Eq.14** that 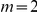, meaning that the first 2-order predictions should be taken into consideration. Substituting these data into **Eq.13**, we obtained the overall success rate by the first two orders predictions for the drugs in 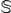 was 73.25%.

These results indicate that the integrated method performed quite well in identifying drugs among their 14 main ATC-classes, and that more attention should be paid to the results hit by the first two order predictions because they covered more than 70% of the true ATC-classes.

Finally, it is instructive to point out that although the above demonstrations were given for identifying query drug compounds among their main (or 1^st^ level) classification, the method developed here can be straightforwardly extended to cover the 2^nd^, 3^rd^, 4^th^, 5^th^ or any lower-level classification as long as the corresponding statistically significant datasets for training the predictor are available.

## Supporting Information

Supporting Information S1 List of the 4,376 drugs in the ATC classification system extracted from KEGG.
(PDF)

Supporting Information S2This dataset 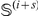 contains 2,138 drugs classified into 14 main ATC classes. Each of the drugs listed here contains both chemical-chemical interaction and chemical-chemical similarity informations. Among the 2,138 different drugs (2,655 virtual drugs), 1,838 belong to one class; 190 to two classes; 57 to three classes, 19 to four classes, 14 to five classes, and 20 to six classes. None of the drugs listed here belongs to seven and more classes.(PDF)
