# Supplementary material for: Predicting Anatomical Therapeutic Chemical (ATC) Classification of Drugs by Integrating Chemical-Chemical Interactions and Similarities
Source: PLoS One. 2012 Apr 13;7(4):e35254. doi: 10.1371/journal.pone.0035254 (PMC3325992; doi:10.1371/journal.pone.0035254)
Supplement: Supporting Information S1 — List of the 4,376 drugs in the ATC classification system extracted from KEGG. (PDF) [file pone.0035254.s001.pdf]

Online Supporting Information S1. This benchmark dataset $\mathbb{S}$ contains 3,833 drug compounds (4,912 virtual compounds), classified into 14 main ATC classes. Among the 3,883 different drugs, 3,295 belong to one class; 370 to two classes; 110 to three classes, 37 to four classes, 27 to five classes, and 44 to six classes. None of the drugs listed here belongs to seven and more classes. See the text and Eqs.1-3 of the paper for further explanation.

(1) $\mathbb{S}_1$: 540 compounds of "Alimentary tract and metabolism"

D00002	D00006	D00008	D00015	D00018	D00028	D00029	D00036
D00037	D00048	D00050	D00062	D00086	D00088	D00095	D00096
D00109	D00113	D00122	D00129	D00138	D00147	D00152	D00157
D00163	D00165	D00170	D00187	D00188	D00193	D00201	D00202
D00203	D00212	D00216	D00219	D00244	D00245	D00246	D00271
D00274	D00282	D00292	D00295	D00299	D00305	D00306	D00307
D00318	D00335	D00336	D00352	D00355	D00367	D00377	D00379
D00380	D00385	D00389	D00395	D00409	D00416	D00419	D00422
D00440	D00444	D00446	D00448	D00455	D00456	D00462	D00472
D00473	D00481	D00490	D00526	D00540	D00593	D00594	D00595
D00596	D00625	D00673	D00677	D00678	D00689	D00717	D00719
D00720	D00721	D00723	D00724	D00725	D00726	D00727	D00729
D00731	D00734	D00850	D00858	D00866	D00868	D00882	D00884
D00926	D00931	D00932	D00934	D00935	D00936	D00937	D00938
D00943	D00944	D00945	D00955	D00956	D00972	D00975	D00976
D00977	D00978	D00980	D00981	D00982	D00983	D00984	D00985
D00996	D01003	D01005	D01006	D01066	D01077	D01081	D01082
D01108	D01111	D01131	D01149	D01165	D01167	D01191	D01201
D01207	D01222	D01225	D01234	D01239	D01255	D01297	D01298
D01301	D01306	D01319	D01345	D01350	D01356	D01357	D01365
D01375	D01377	D01402	D01406	D01410	D01414	D01442	D01446
D01451	D01467	D01476	D01500	D01510	D01518	D01529	D01530
D01532	D01542	D01561	D01584	D01588	D01599	D01612	D01615
D01616	D01619	D01622	D01632	D01637	D01642	D01648	D01650
D01665	D01721	D01732	D01735	D01737	D01742	D01745	D01798
D01813	D01818	D01828	D01854	D01886	D01891	D01899	D01913
D01942	D01948	D01976	D01984	D01998	D02012	D02026	D02030
D02032	D02039	D02041	D02049	D02056	D02057	D02060	D02069
D02071	D02077	D02092	D02094	D02122	D02129	D02130	D02138
D02149	D02156	D02171	D02174	D02176	D02179	D02206	D02213
D02218	D02254	D02255	D02256	D02286	D02288	D02293	D02316
D02323	D02324	D02332	D02390	D02397	D02414	D02416	D02425
D02427	D02430	D02437	D02440	D02447	D02535	D02554	D02571
D02589	D02591	D02592	D02593	D02712	D02715	D02730	D02774
D02807	D02821	D02829	D02859	D02862	D02877	D02878	D02968

D03011	D03088	D03101	D03103	D03262	D03265	D03277	D03281
D03294	D03300	D03301	D03302	D03303	D03309	D03370	D03463
D03477	D03495	D03496	D03503	D03534	D03609	D03634	D03713
D03801	D03809	D03814	D03824	D03827	D03860	D03903	D03904
D04028	D04056	D04148	D04370	D04467	D04479	D04713	D04790
D04834	D04835	D04868	D04893	D04966	D05008	D05010	D05016
D05017	D05032	D05045	D05099	D05116	D05140	D05177	D05181
D05241	D05259	D05261	D05276	D05320	D05321	D05343	D05353
D05456	D05458	D05459	D05463	D05489	D05535	D05578	D05644
D05674	D05699	D05725	D05731	D05853	D05864	D05868	D05869
D05877	D05919	D06056	D06171	D06174	D06177	D06216	D06371
D06389	D06408	D06418	D06419	D06420	D06421	D06423	D06425
D06521	D06522	D06543	D06553	D06574	D06618	D06645	D06877
D06878	D07060	D07066	D07067	D07068	D07069	D07070	D07072
D07073	D07075	D07076	D07078	D07079	D07080	D07081	D07083
D07084	D07087	D07088	D07089	D07091	D07092	D07093	D07094
D07095	D07097	D07098	D07099	D07101	D07102	D07103	D07104
D07105	D07107	D07108	D07109	D07110	D07111	D07113	D07114
D07115	D07117	D07118	D07127	D07128	D07129	D07130	D07175
D07192	D07251	D07425	D07440	D07444	D07456	D07467	D07477
D07488	D07495	D07508	D07516	D07517	D07523	D07524	D07525
D07531	D07539	D07560	D07563	D07575	D07579	D07580	D07581
D07582	D07586	D07587	D07592	D07615	D07627	D07628	D07668
D07669	D07689	D07746	D07753	D07796	D07797	D07798	D07799
D07800	D07801	D07802	D07805	D07820	D07821	D07861	D07867
D07868	D07876	D07877	D07879	D07891	D07917	D07945	D07951
D07994	D08079	D08144	D08160	D08193	D08214	D08238	D08250
D08251	D08260	D08263	D08266	D08295	D08325	D08326	D08351
D08352	D08369	D08378	D08382	D08389	D08401	D08409	D08412
D08413	D08414	D08415	D08416	D08454	D08463	D08464	D08491
D08494	D08495	D08505	D08513	D08515	D08516	D08531	D08580
D08581	D08608	D08610	D08612	D08639	D08903	D08996	D09000
D09205	D09339	D09566	D09739				

(2) $\mathbb{S}_2$: 133 compounds of “Blood and blood forming organs”

D00009	D00011	D00023	D00042	D00060	D00062	D00070	D00095
D00096	D00106	D00109	D00148	D00160	D00166	D00181	D00302
D00396	D00564	D00769	D00856	D00858	D00862	D00870	D00996
D01027	D01028	D01029	D01062	D01108	D01126	D01136	D01139
D01154	D01194	D01203	D01248	D01280	D01282	D01337	D01345
D01525	D01551	D01725	D01766	D01779	D01781	D01844	D01864
D01896	D01981	D02047	D02056	D02057	D02058	D02060	D02149
D02256	D02304	D02335	D02394	D02464	D02707	D02720	D02721
D02774	D03246	D03265	D03383	D03385	D03463	D03798	D03978

D04172	D04834	D04836	D04929	D05140	D05181	D05457	D05597
D05855	D05869	D06213	D06389	D06483	D06539	D06540	D06541
D06888	D07064	D07082	D07086	D07131	D07135	D07136	D07137
D07138	D07139	D07140	D07141	D07142	D07143	D07144	D07145
D07146	D07426	D07510	D07560	D07568	D07579	D07580	D07581
D07582	D07606	D07666	D07668	D07669	D07729	D07736	D07985
D07994	D08015	D08016	D08065	D08177	D08260	D08354	D08594
D08607	D08628	D08682	D09000	D09017			

(3) $\mathbb{S}_3$: 591 compounds of "Cardiovascular system"

D00033	D00045	D00049	D00076	D00088	D00095	D00098	D00112
D00114	D00124	D00126	D00141	D00165	D00178	D00180	D00190
D00197	D00198	D00199	D00231	D00235	D00244	D00247	D00251
D00255	D00272	D00279	D00281	D00286	D00292	D00294	D00297
D00298	D00303	D00313	D00319	D00325	D00331	D00334	D00340
D00345	D00349	D00357	D00358	D00359	D00362	D00378	D00382
D00383	D00385	D00386	D00400	D00405	D00417	D00418	D00421
D00431	D00432	D00434	D00437	D00438	D00443	D00459	D00461
D00472	D00476	D00477	D00483	D00501	D00507	D00509	D00511
D00513	D00515	D00516	D00519	D00522	D00523	D00551	D00552
D00565	D00597	D00598	D00599	D00600	D00601	D00602	D00603
D00604	D00606	D00608	D00609	D00611	D00612	D00613	D00614
D00615	D00616	D00617	D00618	D00619	D00620	D00621	D00622
D00623	D00624	D00626	D00627	D00629	D00630	D00631	D00632
D00633	D00634	D00635	D00636	D00637	D00638	D00639	D00640
D00642	D00643	D00644	D00645	D00647	D00648	D00649	D00650
D00654	D00656	D00657	D00658	D00733	D00739	D00740	D00741
D00762	D00887	D00889	D00892	D00893	D00972	D00975	D00976
D00977	D00978	D00980	D00981	D00982	D00983	D00984	D00985
D00996	D00997	D01018	D01019	D01020	D01026	D01041	D01065
D01069	D01095	D01104	D01115	D01119	D01122	D01135	D01152
D01173	D01182	D01197	D01204	D01208	D01212	D01213	D01220
D01227	D01233	D01236	D01238	D01239	D01240	D01256	D01290
D01302	D01304	D01307	D01320	D01326	D01333	D01357	D01366
D01367	D01369	D01379	D01386	D01390	D01402	D01405	D01419
D01436	D01440	D01442	D01445	D01454	D01455	D01471	D01510
D01543	D01549	D01553	D01565	D01571	D01573	D01603	D01605
D01606	D01615	D01619	D01632	D01634	D01637	D01667	D01673
D01721	D01741	D01748	D01754	D01756	D01794	D01804	D01806
D01810	D01812	D01813	D01822	D01825	D01833	D01840	D01849
D01862	D01877	D01886	D01908	D01915	D01943	D01944	D01948
D01966	D01969	D01972	D01993	D01998	D02032	D02045	D02061
D02066	D02082	D02084	D02085	D02086	D02087	D02088	D02110
D02149	D02150	D02156	D02167	D02174	D02237	D02239	D02258

D02268	D02272	D02276	D02286	D02288	D02300	D02338	D02342
D02356	D02358	D02363	D02383	D02386	D02460	D02537	D02564
D02587	D02591	D02592	D02631	D02690	D02705	D02742	D02769
D02910	D02914	D02939	D02969	D02976	D03115	D03208	D03301
D03363	D03415	D03450	D03471	D03487	D03492	D03507	D03521
D03664	D03750	D03752	D03753	D03765	D03767	D03830	D03879
D03880	D03881	D03891	D03892	D03914	D03991	D03994	D04004
D04018	D04040	D04051	D04079	D04111	D04161	D04195	D04218
D04219	D04221	D04382	D04386	D04398	D04399	D04438	D04467
D04488	D04490	D04492	D04531	D04532	D04657	D04720	D04733
D04774	D04778	D04825	D04897	D04991	D05011	D05024	D05077
D05087	D05093	D05107	D05127	D05158	D05206	D05428	D05442
D05482	D05587	D05606	D05627	D05700	D05701	D05711	D05967
D06010	D06102	D06172	D06216	D06234	D06328	D06329	D06334
D06341	D06392	D06401	D06412	D06534	D06535	D06536	D06537
D06606	D06622	D06646	D06652	D06653	D06665	D06881	D06882
D07073	D07077	D07147	D07148	D07149	D07150	D07151	D07155
D07156	D07157	D07158	D07159	D07160	D07161	D07162	D07164
D07165	D07166	D07167	D07168	D07169	D07170	D07171	D07172
D07173	D07174	D07176	D07177	D07178	D07179	D07180	D07181
D07182	D07183	D07184	D07185	D07186	D07187	D07188	D07189
D07190	D07191	D07427	D07428	D07447	D07450	D07474	D07490
D07494	D07499	D07500	D07509	D07510	D07520	D07526	D07537
D07538	D07546	D07548	D07551	D07555	D07590	D07619	D07624
D07660	D07661	D07697	D07699	D07724	D07748	D07781	D07796
D07797	D07798	D07799	D07800	D07801	D07802	D07830	D07833
D07834	D07843	D07845	D07858	D07870	D07874	D07892	D07894
D07916	D07922	D07931	D07941	D07943	D07946	D07955	D07962
D07973	D07983	D07992	D07999	D08001	D08009	D08020	D08030
D08031	D08037	D08038	D08044	D08057	D08058	D08059	D08060
D08064	D08068	D08074	D08090	D08092	D08093	D08095	D08099
D08106	D08111	D08127	D08131	D08146	D08155	D08180	D08192
D08194	D08201	D08205	D08212	D08215	D08217	D08220	D08225
D08239	D08243	D08244	D08254	D08270	D08271	D08284	D08286
D08308	D08309	D08311	D08318	D08321	D08340	D08358	D08362
D08365	D08366	D08407	D08410	D08411	D08412	D08413	D08414
D08415	D08421	D08422	D08435	D08443	D08453	D08458	D08459
D08482	D08492	D08499	D08517	D08525	D08529	D08566	D08572
D08600	D08614	D08619	D08642	D08662	D08663	D08676	D08677
D08678	D08688	D08735	D08890	D08892	D08966	D09038	

(4) $\mathbb{S}_4$: 421 compounds of “Dermatologicals”

D00008	D00023	D00024	D00033	D00054	D00068	D00073	D00088
D00094	D00097	D00104	D00107	D00108	D00129	D00133	D00137

D00139	D00140	D00156	D00164	D00165	D00193	D00201	D00202
D00205	D00209	D00213	D00222	D00233	D00244	D00246	D00277
D00282	D00290	D00292	D00300	D00316	D00321	D00322	D00323
D00325	D00328	D00339	D00342	D00348	D00351	D00358	D00372
D00381	D00385	D00389	D00407	D00409	D00416	D00418	D00433
D00472	D00480	D00494	D00526	D00551	D00552	D00566	D00592
D00666	D00669	D00689	D00690	D00728	D00733	D00739	D00741
D00751	D00770	D00802	D00810	D00829	D00851	D00857	D00858
D00859	D00861	D00862	D00863	D00864	D00865	D00870	D00881
D00882	D00883	D00884	D00885	D00886	D00890	D00903	D00904
D00935	D00972	D00975	D00976	D00977	D00978	D00979	D00980
D00981	D00982	D00983	D00984	D00985	D01034	D01046	D01047
D01062	D01063	D01072	D01073	D01076	D01082	D01093	D01094
D01108	D01112	D01124	D01125	D01132	D01140	D01143	D01166
D01239	D01242	D01248	D01266	D01272	D01273	D01308	D01327
D01335	D01343	D01345	D01357	D01361	D01364	D01367	D01384
D01387	D01402	D01429	D01442	D01458	D01464	D01472	D01480
D01510	D01516	D01575	D01596	D01615	D01619	D01621	D01632
D01637	D01664	D01702	D01708	D01711	D01720	D01730	D01764
D01768	D01775	D01801	D01820	D01825	D01886	D01905	D01910
D01938	D01948	D01990	D01995	D01998	D02009	D02032	D02053
D02086	D02091	D02122	D02124	D02132	D02143	D02156	D02159
D02164	D02174	D02184	D02185	D02195	D02219	D02255	D02286
D02287	D02288	D02308	D02351	D02375	D02393	D02414	D02419
D02435	D02445	D02456	D02476	D02477	D02500	D02523	D02524
D02525	D02543	D02554	D02557	D02582	D02583	D02589	D02591
D02592	D02754	D02815	D02845	D02905	D02906	D02907	D02923
D03034	D03093	D03101	D03127	D03135	D03145	D03290	D03300
D03301	D03315	D03325	D03360	D03383	D03454	D03463	D03473
D03477	D03488	D03535	D03538	D03541	D03680	D03696	D03697
D03812	D03813	D03854	D03884	D03936	D03956	D04054	D04201
D04208	D04217	D04218	D04219	D04221	D04225	D04281	D04409
D04467	D04624	D04636	D04815	D04926	D04934	D05000	D05001
D05002	D05016	D05017	D05072	D05082	D05140	D05225	D05321
D05322	D05407	D05463	D05465	D05480	D05529	D05601	D05660
D05720	D05815	D05840	D06139	D06216	D06226	D06246	D06315
D06526	D06527	D06534	D06535	D06536	D06537	D06542	D06543
D06661	D06876	D06883	D07067	D07069	D07073	D07116	D07193
D07194	D07195	D07196	D07197	D07198	D07199	D07200	D07201
D07202	D07203	D07204	D07205	D07206	D07207	D07208	D07209
D07210	D07211	D07212	D07213	D07214	D07215	D07492	D07493
D07495	D07521	D07596	D07664	D07666	D07668	D07669	D07672
D07674	D07675	D07676	D07679	D07684	D07689	D07715	D07717
D07719	D07753	D07796	D07797	D07798	D07799	D07800	D07801

D07802	D07813	D07816	D07817	D07818	D07819	D07822	D07827
D07853	D07883	D07909	D07910	D07972	D07973	D07980	D07981
D07994	D08000	D08003	D08013	D08043	D08091	D08127	D08183
D08184	D08214	D08227	D08245	D08260	D08296	D08313	D08319
D08399	D08407	D08412	D08413	D08414	D08415	D08432	D08436
D08462	D08477	D08510	D08535	D08543	D08554	D08556	D08587
D08645	D08652	D08660	D08768	D08894			

(5) $\mathbb{S}_5$: 248 compounds of "Genito-urinary system and sex hormones"

D00010	D00018	D00054	D00066	D00067	D00075	D00079	D00081
D00104	D00105	D00111	D00118	D00126	D00180	D00182	D00185
D00202	D00203	D00205	D00220	D00269	D00277	D00282	D00289
D00312	D00321	D00327	D00351	D00408	D00409	D00416	D00419
D00465	D00554	D00577	D00581	D00585	D00610	D00646	D00680
D00682	D00718	D00722	D00731	D00780	D00830	D00863	D00880
D00881	D00882	D00884	D00885	D00888	D00890	D00898	D00946
D00949	D00950	D00951	D00952	D00953	D00957	D00958	D00959
D00962	D00970	D00987	D01007	D01024	D01043	D01046	D01050
D01072	D01073	D01103	D01122	D01127	D01139	D01148	D01159
D01163	D01217	D01263	D01269	D01281	D01294	D01299	D01348
D01352	D01364	D01368	D01374	D01410	D01413	D01428	D01462
D01480	D01543	D01571	D01575	D01580	D01596	D01617	D01639
D01664	D01692	D01699	D01829	D01873	D01921	D01953	D01965
D01986	D01989	D01990	D01995	D02004	D02008	D02073	D02132
D02143	D02185	D02199	D02217	D02218	D02229	D02256	D02343
D02359	D02367	D02414	D02582	D02583	D02705	D02725	D02731
D03008	D03028	D03062	D03165	D03260	D03269	D03399	D03462
D03488	D03538	D03649	D03654	D03820	D03936	D04021	D04061
D04063	D04064	D04065	D04104	D04157	D04317	D04490	D04624
D04672	D04885	D04947	D05016	D05017	D05274	D05321	D05322
D05450	D05460	D05679	D06085	D06086	D06087	D06425	D06606
D06671	D07109	D07110	D07124	D07193	D07207	D07208	D07216
D07217	D07218	D07219	D07220	D07221	D07222	D07223	D07224
D07225	D07226	D07425	D07430	D07456	D07460	D07516	D07517
D07551	D07598	D07670	D07674	D07675	D07726	D07766	D07826
D07905	D07906	D07918	D07919	D07920	D07921	D07928	D07939
D07961	D08052	D08053	D08058	D08059	D08132	D08166	D08167
D08207	D08214	D08239	D08281	D08285	D08296	D08313	D08346
D08431	D08441	D08457	D08465	D08514	D08522	D08532	D08560
D08569	D08573	D08574	D08668	D08685	D08923	D09567	D09687

(6) $\mathbb{S}_6$: 126 compounds of "Systemic hormonal preparations, excluding sex hormones and insulins"

D00088	D00089	D00101	D00165	D00244	D00249	D00284	D00291
D00292	D00366	D00385	D00401	D00407	D00442	D00472	D00473
D00562	D00751	D00930	D00972	D00973	D00975	D00976	D00977
D00978	D00979	D00980	D00981	D00982	D00983	D00984	D00985
D00986	D00988	D00990	D01009	D01010	D01011	D01180	D01229
D01239	D01357	D01402	D01442	D01510	D01615	D01619	D01632
D01637	D01685	D01886	D01948	D01998	D02032	D02105	D02116
D02117	D02156	D02174	D02235	D02250	D02286	D02288	D02369
D02591	D02592	D02983	D03267	D03301	D03504	D03505	D03561
D03594	D03671	D03698	D03699	D04218	D04219	D04302	D04361
D04467	D05000	D05001	D05002	D05230	D05729	D06216	D06281
D06495	D06672	D06673	D07073	D07203	D07214	D07227	D07228
D07229	D07230	D07231	D07232	D07464	D07519	D07616	D07665
D07749	D07792	D07796	D07797	D07798	D07799	D07800	D07801
D07802	D07967	D07973	D08010	D08027	D08241	D08412	D08413
D08414	D08415	D08416	D08509	D08571	D08659		

(7) $\mathbb{S}_7$: 521 compounds of "Antiinfectives for systemic use"

D00063	D00104	D00135	D00140	D00144	D00145	D00162	D00183
D00186	D00201	D00203	D00204	D00205	D00210	D00211	D00212
D00213	D00222	D00223	D00229	D00230	D00240	D00256	D00257
D00258	D00259	D00260	D00261	D00262	D00263	D00264	D00273
D00276	D00277	D00278	D00285	D00290	D00296	D00307	D00310
D00317	D00322	D00323	D00333	D00342	D00344	D00346	D00350
D00351	D00353	D00393	D00398	D00406	D00409	D00412	D00413
D00416	D00423	D00424	D00427	D00429	D00433	D00435	D00439
D00445	D00447	D00450	D00453	D00466	D00497	D00578	D00579
D00587	D00588	D00589	D00590	D00591	D00592	D00660	D00849
D00850	D00851	D00854	D00855	D00856	D00865	D00866	D00867
D00869	D00870	D00872	D00873	D00874	D00875	D00877	D00878
D00879	D00882	D00891	D00894	D00895	D00896	D00897	D00899
D00900	D00901	D00902	D00905	D00907	D00908	D00909	D00910
D00911	D00912	D00913	D00914	D00915	D00916	D00917	D00918
D00919	D00920	D00921	D00922	D00923	D00924	D00925	D00926
D00927	D00929	D00947	D01047	D01052	D01053	D01057	D01063
D01066	D01072	D01073	D01074	D01075	D01078	D01079	D01080
D01142	D01144	D01153	D01157	D01160	D01178	D01195	D01199
D01216	D01235	D01249	D01251	D01262	D01276	D01283	D01322
D01339	D01350	D01361	D01401	D01407	D01415	D01425	D01426
D01429	D01431	D01486	D01489	D01499	D01501	D01517	D01523
D01526	D01528	D01531	D01596	D01628	D01629	D01636	D01649
D01655	D01668	D01680	D01710	D01716	D01739	D01753	D01819

D01826	D01836	D01863	D01897	D01904	D01937	D01954	D01982
D01990	D01995	D02002	D02005	D02009	D02049	D02065	D02119
D02121	D02122	D02123	D02124	D02129	D02132	D02134	D02136
D02137	D02138	D02142	D02143	D02157	D02178	D02181	D02184
D02185	D02186	D02187	D02188	D02190	D02191	D02196	D02198
D02199	D02201	D02203	D02209	D02216	D02221	D02222	D02223
D02226	D02228	D02231	D02233	D02241	D02251	D02255	D02267
D02282	D02297	D02299	D02301	D02302	D02305	D02306	D02307
D02318	D02336	D02339	D02344	D02345	D02346	D02348	D02352
D02353	D02376	D02406	D02420	D02434	D02435	D02436	D02439
D02450	D02465	D02469	D02471	D02474	D02475	D02495	D02496
D02497	D02501	D02503	D02505	D02506	D02508	D02509	D02516
D02517	D02521	D02523	D02524	D02525	D02540	D02541	D02542
D02543	D02544	D02545	D02549	D02555	D02711	D02734	D02861
D02867	D02888	D02889	D02925	D03039	D03211	D03256	D03262
D03368	D03387	D03424	D03426	D03428	D03431	D03477	D03537
D03571	D03640	D03656	D03680	D03705	D03706	D03707	D03833
D03835	D03837	D03843	D03865	D03895	D03903	D03904	D04008
D04020	D04049	D04054	D04112	D04196	D04253	D04254	D04281
D04301	D04859	D04972	D05016	D05017	D05021	D05022	D05026
D05045	D05110	D05140	D05271	D05274	D05322	D05351	D05382
D05406	D05407	D05408	D05411	D05460	D05461	D05520	D05521
D05528	D05908	D05949	D05957	D05972	D06057	D06064	D06137
D06138	D06236	D06298	D06299	D06300	D06390	D06478	D06558
D06670	D06675	D06676	D06884	D06885	D07057	D07133	D07199
D07233	D07234	D07235	D07236	D07237	D07238	D07239	D07240
D07241	D07242	D07243	D07244	D07245	D07246	D07247	D07248
D07249	D07250	D07433	D07452	D07462	D07471	D07484	D07486
D07487	D07570	D07607	D07614	D07621	D07626	D07629	D07635
D07636	D07637	D07638	D07639	D07640	D07641	D07643	D07644
D07645	D07647	D07648	D07649	D07650	D07651	D07653	D07654
D07656	D07658	D07659	D07674	D07675	D07689	D07702	D07710
D07733	D07735	D07746	D07782	D07811	D07876	D07877	D07896
D07908	D07909	D07910	D07925	D07965	D07994	D07995	D08000
D08003	D08011	D08012	D08013	D08017	D08109	D08120	D08143
D08185	D08191	D08199	D08214	D08218	D08237	D08259	D08260
D08268	D08287	D08291	D08306	D08307	D08337	D08350	D08360
D08379	D08380	D08396	D08401	D08438	D08478	D08479	D08480
D08483	D08496	D08526	D08527	D08528	D08531	D08533	D08534
D08538	D08539	D08540	D08543	D08557	D08563	D08567	D08582
D08583	D08593	D08605	D08644	D08652	D08654	D08664	D08884
D08886							

(8) $\mathbb{S}_8$: 232 compounds of "Antineoplastic and immunomodulating agents"

D00094	D00107	D00125	D00142	D00155	D00161	D00168	D00184
D00208	D00214	D00238	D00248	D00254	D00266	D00275	D00287
D00288	D00341	D00343	D00363	D00369	D00420	D00467	D00468
D00478	D00491	D00554	D00567	D00573	D00574	D00577	D00583
D00584	D00586	D00749	D00752	D00753	D00754	D00946	D00951
D00952	D00960	D00961	D00963	D00964	D00965	D00966	D00967
D00989	D01059	D01061	D01064	D01068	D01155	D01159	D01161
D01223	D01244	D01264	D01270	D01275	D01363	D01370	D01441
D01516	D01566	D01695	D01747	D01760	D01769	D01784	D01790
D01831	D01885	D01907	D01911	D01935	D01977	D02020	D02106
D02115	D02131	D02165	D02166	D02168	D02177	D02197	D02214
D02321	D02368	D02398	D02494	D02697	D02698	D02714	D02738
D02747	D02748	D02756	D02815	D02841	D02908	D02933	D03021
D03033	D03046	D03106	D03150	D03229	D03546	D03637	D03658
D03665	D03786	D03828	D03899	D03961	D03962	D04023	D04024
D04066	D04107	D04187	D04405	D04444	D04645	D04687	D04862
D04872	D04931	D04988	D05094	D05095	D05096	D05134	D05333
D05380	D05522	D05589	D05602	D05756	D05807	D05822	D05932
D06066	D06067	D06068	D06109	D06117	D06130	D06199	D06247
D06248	D06272	D06304	D06320	D06386	D06397	D06402	D06407
D06413	D06414	D06488	D06503	D06619	D06637	D07071	D07085
D07100	D07252	D07253	D07254	D07255	D07256	D07257	D07258
D07259	D07260	D07261	D07434	D07455	D07472	D07501	D07535
D07567	D07671	D07760	D07772	D07776	D07826	D07866	D07901
D07907	D07928	D07966	D07974	D08032	D08062	D08066	D08086
D08108	D08113	D08166	D08167	D08173	D08204	D08224	D08276
D08386	D08423	D08524	D08552	D08556	D08559	D08603	D08618
D08620	D08649	D08675	D08679	D08680	D08854	D08901	D08914
D08953	D08956	D08971	D09032	D09033	D09654	D09724	D09733

(9) $\mathbb{S}_9$: 208 compounds of “Musculo-skeletal system”

D00080	D00118	D00120	D00126	D00127	D00132	D00141	D00151
D00158	D00169	D00224	D00237	D00241	D00250	D00268	D00314
D00315	D00330	D00402	D00425	D00449	D00452	D00463	D00475
D00492	D00496	D00510	D00567	D00568	D00570	D00755	D00758
D00759	D00760	D00763	D00764	D00765	D00766	D00767	D00768
D00771	D00772	D00774	D00776	D00813	D00903	D00904	D00939
D00941	D00942	D00968	D00969	D00970	D00991	D00992	D00997
D01043	D01049	D01056	D01122	D01183	D01206	D01215	D01252
D01271	D01289	D01305	D01325	D01338	D01344	D01410	D01437
D01475	D01507	D01513	D01545	D01565	D01567	D01581	D01582
D01594	D01671	D01675	D01765	D01767	D01809	D01823	D01824
D01841	D01866	D01968	D01975	D02064	D02110	D02207	D02274

D02275	D02285	D02290	D02292	D02341	D02347	D02350	D02355
D02373	D02595	D02709	D02966	D03080	D03234	D03254	D03544
D03545	D03710	D03714	D03715	D03716	D03717	D03718	D04078
D04102	D04334	D04435	D04486	D04490	D04530	D05078	D05319
D05511	D05512	D05513	D05515	D05638	D06072	D06073	D06378
D06379	D06382	D06388	D06606	D07119	D07215	D07219	D07262
D07263	D07264	D07265	D07266	D07267	D07268	D07269	D07270
D07271	D07272	D07273	D07274	D07275	D07276	D07277	D07278
D07279	D07280	D07281	D07503	D07516	D07517	D07549	D07564
D07720	D07758	D07816	D07817	D07818	D07819	D07898	D07953
D08022	D08023	D08043	D08056	D08058	D08059	D08063	D08102
D08103	D08104	D08142	D08162	D08265	D08267	D08275	D08324
D08330	D08363	D08364	D08418	D08419	D08427	D08468	D08484
D08521	D08599	D08611	D08614	D08617	D08655	D08689	D09568

(10) $\mathbb{S}_{10}$: 737 compounds of "Nervous system"

D00020	D00033	D00057	D00058	D00059	D00102	D00109	D00110
D00130	D00131	D00136	D00138	D00217	D00225	D00228	D00250
D00252	D00253	D00265	D00267	D00270	D00280	D00281	D00283
D00293	D00304	D00308	D00311	D00320	D00326	D00329	D00332
D00338	D00354	D00358	D00365	D00370	D00373	D00374	D00375
D00376	D00387	D00388	D00390	D00392	D00394	D00399	D00403
D00404	D00415	D00426	D00428	D00430	D00451	D00454	D00457
D00458	D00464	D00470	D00474	D00479	D00482	D00484	D00487
D00493	D00495	D00498	D00499	D00500	D00502	D00503	D00504
D00505	D00506	D00508	D00512	D00514	D00524	D00525	D00528
D00530	D00531	D00532	D00533	D00535	D00536	D00537	D00538
D00539	D00542	D00543	D00544	D00545	D00546	D00547	D00548
D00549	D00550	D00551	D00552	D00553	D00555	D00556	D00557
D00559	D00560	D00561	D00563	D00566	D00569	D00604	D00661
D00670	D00672	D00674	D00675	D00676	D00679	D00681	D00693
D00694	D00695	D00696	D00698	D00700	D00701	D00702	D00704
D00705	D00706	D00708	D00709	D00710	D00711	D00712	D00714
D00732	D00733	D00735	D00737	D00738	D00740	D00741	D00744
D00775	D00777	D00778	D00779	D00780	D00781	D00782	D00784
D00785	D00786	D00787	D00788	D00789	D00790	D00791	D00792
D00793	D00794	D00795	D00796	D00797	D00798	D00799	D00800
D00801	D00809	D00810	D00811	D00812	D00814	D00815	D00816
D00817	D00818	D00819	D00820	D00821	D00822	D00823	D00824
D00825	D00826	D00835	D00836	D00837	D00838	D00839	D00842
D00843	D00845	D00846	D00847	D00987	D00993	D00995	D00998
D01000	D01001	D01044	D01071	D01096	D01101	D01105	D01107
D01110	D01116	D01118	D01150	D01164	D01177	D01179	D01190

D01205	D01219	D01226	D01228	D01230	D01243	D01245	D01253
D01254	D01267	D01268	D01285	D01287	D01292	D01293	D01295
D01296	D01303	D01310	D01314	D01321	D01328	D01351	D01354
D01355	D01358	D01371	D01372	D01380	D01382	D01383	D01391
D01399	D01408	D01412	D01437	D01447	D01448	D01450	D01451
D01453	D01462	D01465	D01466	D01477	D01481	D01482	D01485
D01514	D01520	D01522	D01537	D01546	D01548	D01592	D01597
D01630	D01657	D01740	D01744	D01750	D01762	D01772	D01776
D01787	D01793	D01807	D01811	D01832	D01860	D01871	D01876
D01883	D01898	D01901	D01902	D01914	D01930	D01939	D01973
D02004	D02022	D02037	D02068	D02071	D02074	D02078	D02086
D02095	D02096	D02097	D02100	D02102	D02103	D02111	D02135
D02153	D02154	D02155	D02160	D02162	D02163	D02173	D02175
D02182	D02200	D02208	D02211	D02227	D02236	D02242	D02243
D02246	D02247	D02248	D02252	D02253	D02260	D02271	D02340
D02357	D02360	D02361	D02362	D02408	D02536	D02558	D02559
D02561	D02562	D02565	D02566	D02567	D02570	D02572	D02573
D02574	D02575	D02578	D02579	D02580	D02599	D02604	D02608
D02609	D02611	D02612	D02613	D02618	D02619	D02621	D02622
D02623	D02624	D02625	D02626	D02627	D02629	D02630	D02642
D02643	D02671	D02680	D02682	D02683	D02689	D02716	D02717
D02766	D02770	D02771	D02780	D02787	D02817	D02824	D02825
D02941	D02942	D02991	D02995	D03089	D03102	D03165	D03197
D03199	D03264	D03274	D03276	D03288	D03365	D03385	D03528
D03556	D03562	D03711	D03721	D03731	D03740	D03783	D03785
D03822	D03825	D03852	D03975	D04034	D04038	D04048	D04087
D04088	D04095	D04105	D04127	D04147	D04226	D04257	D04264
D04292	D04314	D04605	D04650	D04716	D04741	D04747	D04749
D04750	D04765	D04882	D04905	D04924	D04965	D04985	D04999
D05028	D05039	D05040	D05113	D05133	D05156	D05181	D05200
D05290	D05312	D05339	D05340	D05374	D05375	D05462	D05478
D05523	D05575	D05592	D05593	D05621	D05623	D05626	D05740
D05768	D05775	D05928	D05938	D06007	D06147	D06168	D06282
D06327	D06338	D06339	D06367	D06534	D06535	D06536	D06537
D06573	D06623	D06887	D07058	D07065	D07122	D07132	D07282
D07283	D07284	D07285	D07286	D07287	D07288	D07289	D07290
D07291	D07292	D07293	D07294	D07295	D07296	D07297	D07298
D07299	D07300	D07301	D07302	D07303	D07304	D07305	D07306
D07307	D07308	D07309	D07310	D07311	D07312	D07313	D07314
D07315	D07316	D07317	D07318	D07319	D07320	D07321	D07322
D07323	D07324	D07325	D07326	D07327	D07328	D07329	D07330
D07331	D07332	D07333	D07335	D07336	D07337	D07338	D07339
D07340	D07341	D07342	D07343	D07344	D07345	D07346	D07347
D07348	D07349	D07350	D07441	D07445	D07448	D07460	D07468

D07473	D07511	D07522	D07528	D07552	D07579	D07580	D07581
D07582	D07589	D07591	D07593	D07601	D07603	D07667	D07678
D07685	D07686	D07687	D07704	D07705	D07725	D07727	D07734
D07756	D07757	D07777	D07778	D07779	D07791	D07793	D07804
D07806	D07808	D07809	D07810	D07812	D07831	D07832	D07836
D07837	D07838	D07854	D07865	D07869	D07872	D07875	D07880
D07881	D07887	D07906	D07913	D07930	D07938	D07944	D07971
D07976	D07977	D07978	D07979	D07984	D07993	D07997	D08035
D08047	D08054	D08070	D08071	D08085	D08098	D08100	D08101
D08116	D08127	D08130	D08132	D08133	D08134	D08135	D08136
D08137	D08140	D08141	D08145	D08148	D08157	D08170	D08171
D08172	D08174	D08181	D08182	D08187	D08190	D08195	D08209
D08216	D08222	D08226	D08233	D08234	D08235	D08246	D08255
D08257	D08258	D08261	D08273	D08280	D08283	D08288	D08297
D08312	D08339	D08341	D08342	D08343	D08349	D08356	D08376
D08385	D08390	D08397	D08405	D08422	D08425	D08426	D08430
D08434	D08439	D08446	D08447	D08456	D08469	D08472	D08473
D08485	D08489	D08490	D08549	D08555	D08575	D08585	D08588
D08589	D08590	D08597	D08606	D08623	D08625	D08626	D08634
D08636	D08637	D08638	D08653	D08667	D08669	D08670	D08673
D08674	D08687	D08690	D08691	D08692	D08693	D09215	D09216
D09569							

(11) $\mathbb{S}_{11}$: 127 compounds of “Antiparasitic products, insecticides and repellents”

D00131	D00134	D00236	D00360	D00368	D00372	D00409	D00436
D00460	D00471	D00486	D00488	D00489	D00534	D00582	D00750
D00802	D00803	D00804	D00805	D00806	D00807	D00808	D00828
D00829	D00831	D00832	D00833	D00834	D00862	D01138	D01393
D01426	D01663	D02114	D02125	D02126	D02127	D02145	D02261
D02262	D02366	D02379	D02387	D02448	D02466	D02472	D02480
D02481	D02482	D02483	D02484	D02485	D02486	D02487	D02489
D02491	D02548	D02922	D03028	D03135	D03469	D03538	D03623
D03985	D04140	D04200	D04895	D05016	D05017	D05170	D05274
D05443	D05486	D05657	D05681	D06051	D06114	D06224	D06238
D06239	D07106	D07109	D07110	D07208	D07212	D07351	D07352
D07353	D07354	D07355	D07356	D07357	D07358	D07359	D07360
D07361	D07362	D07363	D07364	D07365	D07366	D07367	D07368
D07430	D07438	D07530	D07680	D07761	D07763	D07785	D07825
D07844	D07883	D08033	D08050	D08114	D08179	D08214	D08357
D08381	D08420	D08428	D08451	D08460	D08461	D08551	

(12) $\mathbb{S}_{12}$: 427 compounds of “Respiratory system”

D00033	D00062	D00074	D00095	D00110	D00124	D00164	D00175
D00195	D00221	D00227	D00234	D00242	D00244	D00246	D00292
D00300	D00324	D00330	D00337	D00358	D00364	D00371	D00385
D00411	D00472	D00480	D00485	D00494	D00511	D00521	D00526
D00527	D00529	D00552	D00659	D00662	D00663	D00664	D00665
D00666	D00668	D00669	D00671	D00683	D00684	D00685	D00686
D00687	D00688	D00689	D00690	D00691	D00735	D00743	D00756
D00757	D00848	D00857	D00858	D00863	D00972	D00975	D00980
D00981	D00982	D00983	D00984	D00985	D00996	D01016	D01021
D01022	D01023	D01036	D01062	D01076	D01106	D01117	D01123
D01130	D01140	D01143	D01172	D01174	D01192	D01224	D01239
D01242	D01260	D01288	D01317	D01324	D01332	D01336	D01343
D01345	D01347	D01349	D01357	D01360	D01362	D01373	D01385
D01386	D01389	D01390	D01402	D01427	D01428	D01430	D01459
D01475	D01478	D01479	D01490	D01495	D01506	D01510	D01569
D01575	D01576	D01608	D01609	D01615	D01621	D01627	D01632
D01635	D01637	D01700	D01703	D01708	D01712	D01713	D01717
D01771	D01773	D01778	D01782	D01786	D01801	D01828	D01872
D01929	D01946	D01948	D01957	D01978	D01998	D02017	D02023
D02032	D02066	D02086	D02089	D02090	D02091	D02101	D02148
D02149	D02150	D02151	D02156	D02172	D02174	D02182	D02195
D02205	D02212	D02230	D02234	D02245	D02286	D02290	D02327
D02354	D02396	D02404	D02414	D02419	D02589	D02591	D02592
D02610	D02732	D02760	D02822	D02884	D02950	D03051	D03166
D03184	D03290	D03301	D03325	D03360	D03402	D03454	D03463
D03535	D03557	D03580	D03585	D03621	D03622	D03693	D03704
D03725	D03742	D03854	D03888	D03898	D04018	D04080	D04089
D04157	D04163	D04214	D04441	D04625	D04979	D04980	D05006
D05037	D05129	D05130	D05140	D05277	D05321	D05366	D05429
D05484	D05649	D05660	D05718	D05730	D05744	D05792	D06103
D06104	D06107	D06171	D06216	D06246	D06315	D06393	D06534
D06535	D06536	D06537	D06543	D07062	D07073	D07125	D07126
D07194	D07195	D07197	D07198	D07206	D07208	D07370	D07371
D07372	D07373	D07374	D07375	D07376	D07377	D07378	D07379
D07380	D07381	D07382	D07383	D07384	D07385	D07386	D07387
D07388	D07389	D07390	D07391	D07392	D07393	D07394	D07395
D07396	D07397	D07398	D07399	D07400	D07401	D07402	D07403
D07404	D07405	D07406	D07407	D07408	D07409	D07410	D07442
D07458	D07459	D07482	D07483	D07489	D07491	D07492	D07493
D07495	D07512	D07534	D07542	D07543	D07547	D07554	D07557
D07594	D07617	D07618	D07662	D07664	D07666	D07668	D07669
D07672	D07679	D07684	D07713	D07716	D07721	D07740	D07753
D07765	D07790	D07796	D07797	D07798	D07799	D07800	D07801
D07802	D07803	D07847	D07848	D07850	D07853	D07862	D07873

D07878	D07881	D07900	D07902	D07923	D07929	D07949	D07958
D07981	D07990	D07994	D08039	D08043	D08045	D08046	D08088
D08090	D08091	D08105	D08117	D08118	D08119	D08127	D08161
D08163	D08183	D08184	D08227	D08229	D08253	D08260	D08293
D08300	D08310	D08316	D08317	D08322	D08334	D08335	D08353
D08355	D08365	D08366	D08368	D08387	D08408	D08412	D08413
D08414	D08415	D08417	D08424	D08432	D08449	D08474	D08477
D08497	D08520	D08570	D08578	D08579	D08587	D08604	D08610
D08624	D08629	D08645	D08648	D08658	D08684	D08768	D08966
D09318	D09319	D09570					

(13) $\mathbb{S}_{13}$: 390 compounds of “Sensory organs”

D00008	D00010	D00018	D00043	D00054	D00063	D00088	D00095
D00097	D00104	D00110	D00113	D00124	D00127	D00133	D00138
D00140	D00141	D00164	D00165	D00184	D00186	D00193	D00196
D00201	D00204	D00205	D00210	D00213	D00218	D00221	D00222
D00244	D00281	D00292	D00317	D00330	D00333	D00342	D00356
D00358	D00378	D00385	D00391	D00397	D00406	D00416	D00433
D00450	D00453	D00472	D00511	D00518	D00524	D00525	D00526
D00527	D00551	D00566	D00588	D00589	D00598	D00599	D00603
D00604	D00652	D00653	D00655	D00659	D00667	D00728	D00733
D00740	D00741	D00743	D00756	D00757	D00810	D00813	D00851
D00858	D00862	D00863	D00865	D00866	D00867	D00870	D00871
D00873	D00874	D00882	D00884	D00903	D00904	D00955	D00956
D00972	D00973	D00975	D00976	D00977	D00978	D00980	D00981
D00982	D00983	D00984	D00985	D00995	D00996	D00998	D00999
D01002	D01004	D01008	D01016	D01021	D01022	D01023	D01025
D01037	D01053	D01063	D01066	D01072	D01077	D01089	D01162
D01192	D01196	D01239	D01251	D01261	D01273	D01332	D01345
D01357	D01361	D01367	D01386	D01402	D01442	D01451	D01452
D01475	D01476	D01510	D01512	D01536	D01565	D01578	D01594
D01596	D01615	D01619	D01621	D01632	D01637	D01689	D01713
D01717	D01724	D01730	D01733	D01737	D01768	D01802	D01820
D01821	D01825	D01886	D01948	D01954	D01964	D01976	D01995
D01998	D02009	D02024	D02032	D02069	D02070	D02071	D02075
D02076	D02081	D02086	D02110	D02119	D02122	D02133	D02134
D02149	D02156	D02157	D02174	D02182	D02184	D02185	D02193
D02200	D02205	D02216	D02237	D02249	D02255	D02286	D02288
D02289	D02290	D02318	D02336	D02349	D02374	D02393	D02418
D02512	D02523	D02524	D02525	D02541	D02542	D02543	D02549
D02589	D02591	D02592	D02706	D02724	D02750	D02842	D03011
D03163	D03243	D03262	D03301	D03463	D03477	D03538	D03648
D03696	D03814	D03826	D03945	D03946	D03947	D04018	D04054

D04221	D04244	D04281	D04301	D04382	D04467	D04488	D04760
D04762	D04934	D05116	D05129	D05130	D05140	D05143	D05319
D05322	D05408	D05478	D05511	D05512	D05513	D05729	D05762
D05763	D05840	D05872	D05947	D05957	D06216	D06274	D06298
D06299	D06300	D06390	D06543	D07073	D07116	D07148	D07204
D07205	D07206	D07374	D07411	D07412	D07414	D07415	D07461
D07477	D07483	D07486	D07496	D07503	D07510	D07526	D07540
D07541	D07554	D07570	D07624	D07668	D07669	D07674	D07675
D07689	D07702	D07717	D07749	D07753	D07759	D07775	D07796
D07797	D07798	D07799	D07800	D07801	D07802	D07813	D07816
D07817	D07818	D07819	D07840	D07871	D07890	D07900	D07909
D07910	D07929	D07994	D08000	D08003	D08011	D08013	D08030
D08043	D08057	D08104	D08105	D08115	D08117	D08120	D08127
D08139	D08147	D08219	D08237	D08250	D08251	D08253	D08260
D08261	D08268	D08287	D08291	D08293	D08308	D08309	D08319
D08322	D08324	D08365	D08366	D08376	D08401	D08412	D08413
D08414	D08415	D08422	D08436	D08440	D08448	D08477	D08480
D08578	D08600	D08661	D08684	D08872	D08966		

(14) $\mathbb{S}_{14}$: 211 compounds of “Various”

D00003	D00004	D00009	D00014	D00019	D00052	D00068	D00083
D00096	D00114	D00167	D00176	D00196	D00221	D00226	D00294
D00380	D00410	D00469	D00509	D00517	D00571	D00697	D00707
D00988	D01013	D01014	D01015	D01016	D01027	D01060	D01091
D01099	D01108	D01137	D01181	D01186	D01187	D01200	D01211
D01258	D01311	D01312	D01313	D01340	D01346	D01376	D01388
D01421	D01459	D01474	D01555	D01563	D01570	D01572	D01631
D01644	D01645	D01646	D01707	D01714	D01719	D01729	D01755
D01761	D01774	D01797	D01802	D01817	D01843	D01880	D01884
D01893	D01936	D01940	D01979	D01983	D01999	D02006	D02007
D02015	D02021	D02025	D02029	D02036	D02053	D02108	D02117
D02161	D02210	D02240	D02259	D02284	D02312	D02402	D02409
D02418	D02457	D02464	D02707	D03263	D03267	D03308	D03355
D03442	D03443	D03603	D03669	D03670	D03730	D03768	D03770
D03826	D03902	D03943	D03944	D03945	D03946	D03947	D04220
D04283	D04284	D04286	D04288	D04291	D04357	D04361	D04420
D04445	D04559	D04560	D04562	D04563	D04567	D04568	D04570
D04571	D04572	D04573	D04574	D04577	D04582	D04586	D04595
D04596	D04598	D04599	D04604	D04656	D04667	D04715	D04848
D04929	D04983	D05019	D05189	D05215	D05328	D05421	D05436
D05439	D05590	D05647	D05795	D05845	D05860	D05865	D05870
D05940	D05962	D06027	D06029	D06030	D06034	D06039	D06042
D06049	D06177	D06178	D06179	D06338	D06339	D06542	D06890

D06891	D07416	D07418	D07420	D07554	D07780	D07807	D07852
D07934	D07935	D08005	D08006	D08008	D08018	D08025	D08027
D08084	D08247	D08248	D08249	D08262	D08362	D08504	D08509
D08512	D08548	D09213					
