# Supplementary material for: Predicting Anatomical Therapeutic Chemical (ATC) Classification of Drugs by Integrating Chemical-Chemical Interactions and Similarities
Source: PLoS One. 2012 Apr 13;7(4):e35254. doi: 10.1371/journal.pone.0035254 (PMC3325992; doi:10.1371/journal.pone.0035254)
Supplement: Supporting Information S2 — This dataset contains 2,138 drugs classified into 14 main ATC classes. Each of the drugs listed here contains both chemical-chemical interaction and chemical-chemical similarity informations. Among the 2,138 different drugs (2,655 virtual drugs), 1,838 belong to one class; 190 to two classes; 57 to three classes, 19 to four classes, 14 to five classes, and 20 to six classes. None of the drugs listed here belongs to seven and more classes. (PDF) [file pone.0035254.s002.pdf]

Online Supporting Information S2. This dataset $\mathbb{S}^{(i+s)}$ contains 2,138 drugs classified into 14 main ATC classes. Each of the drugs listed here contains both chemical-chemical interaction and chemical-chemical similarity informations. Among the 2,138 different drugs (2,655 virtual drugs), 1,838 belong to one class; 190 to two classes; 57 to three classes, 19 to four classes, 14 to five classes, and 20 to six classes. None of the drugs listed here belongs to seven and more classes.

(1) $\mathbb{S}_1^{(i+s)}$: 282 compounds of "Alimentary tract and metabolism"

D00002 D00008 D00015 D00028 D00029 D00036 D00037 D00048
D00050 D00086 D00088 D00096 D00109 D00122 D00129 D00152
D00165 D00170 D00187 D00188 D00202 D00203 D00216 D00219
D00245 D00246 D00271 D00282 D00292 D00299 D00306 D00318
D00335 D00336 D00352 D00355 D00367 D00377 D00379 D00385
D00389 D00395 D00419 D00440 D00444 D00448 D00462 D00472
D00473 D00481 D00490 D00540 D00593 D00594 D00596 D00625
D00673 D00678 D00689 D00720 D00721 D00723 D00734 D00858
D00882 D00884 D00926 D00931 D00932 D00955 D00956 D00975
D00976 D00977 D00980 D00981 D00982 D00983 D00984 D00985
D01003 D01005 D01108 D01111 D01131 D01165 D01167 D01191
D01201 D01222 D01225 D01234 D01255 D01301 D01306 D01319
D01356 D01365 D01375 D01377 D01406 D01410 D01414 D01446
D01451 D01467 D01500 D01510 D01518 D01529 D01530 D01532
D01542 D01561 D01584 D01588 D01599 D01612 D01615 D01616
D01619 D01637 D01642 D01665 D01721 D01735 D01742 D01745
D01798 D01813 D01818 D01828 D01854 D01886 D01891 D01899
D01913 D01942 D01948 D01976 D01998 D02012 D02032 D02039
D02057 D02071 D02077 D02092 D02129 D02130 D02156 D02171
D02206 D02254 D02256 D02286 D02288 D02323 D02332 D02390
D02397 D02425 D02427 D02430 D02437 D02440 D02535 D02554
D02589 D02715 D02774 D02821 D02829 D02859 D02862 D02877
D02878 D02968 D03011 D03088 D03101 D03262 D03265 D03281
D03294 D03302 D03309 D03463 D03496 D03503 D03534 D03634
D03809 D03814 D03824 D03827 D04028 D04370 D04467 D04479
D04713 D04790 D04834 D04868 D04893 D04966 D05008 D05010
D05016 D05017 D05032 D05045 D05099 D05140 D05177 D05181
D05241 D05261 D05320 D05321 D05343 D05353 D05456 D05459
D05489 D05535 D05578 D05644 D05674 D05699 D05725 D05731
D05853 D05864 D05868 D05877 D05919 D06056 D06171 D06174
D06177 D06371 D06408 D06425 D06522 D06543 D06574 D06618
D06645 D07060 D07107 D07130 D07425 D07444 D07456 D07495
D07523 D07668 D07689 D07753 D07796 D07820 D07861 D07867
D07876 D07945 D08079 D08144 D08250 D08295 D08325 D08351
D08378 D08389 D08409 D08416 D08454 D08463 D08513 D08515
D08531 D08580

(2) $\mathbb{S}_2^{(i+s)}$: 63 compounds of "Blood and blood forming organs"

D00011 D00042 D00070 D00096 D00109 D00148 D00160 D00166
D00181 D00302 D00396 D00769 D00856 D00858 D00862 D00870
D01027 D01062 D01108 D01136 D01139 D01154 D01194 D01248
D01282 D01337 D01525 D01766 D01779 D01781 D01844 D01864
D01896 D01981 D02057 D02256 D02335 D02394 D02720 D02721
D02774 D03246 D03265 D03383 D03385 D03463 D03798 D04172
D04834 D04836 D04929 D05140 D05181 D05457 D05597 D05855
D06213 D06483 D07668 D08354 D08594 D08607 D08682

(3) $\mathbb{S}_3^{(i+s)}$: 325 compounds of “Cardiovascular system”

D00049 D00088 D00112 D00114 D00165 D00178 D00180 D00197
D00198 D00199 D00231 D00247 D00251 D00272 D00279 D00286
D00292 D00294 D00297 D00298 D00319 D00325 D00331 D00334
D00340 D00349 D00359 D00362 D00382 D00385 D00386 D00400
D00418 D00421 D00431 D00432 D00434 D00437 D00438 D00443
D00461 D00472 D00476 D00501 D00509 D00513 D00515 D00516
D00522 D00523 D00552 D00565 D00604 D00611 D00612 D00614
D00615 D00618 D00624 D00626 D00627 D00629 D00630 D00637
D00642 D00643 D00645 D00647 D00650 D00654 D00656 D00657
D00658 D00741 D00762 D00975 D00976 D00977 D00980 D00981
D00982 D00983 D00984 D00985 D01065 D01069 D01095 D01104
D01115 D01119 D01122 D01135 D01152 D01173 D01182 D01197
D01204 D01208 D01212 D01213 D01220 D01227 D01233 D01236
D01238 D01240 D01256 D01290 D01304 D01320 D01333 D01366
D01367 D01369 D01379 D01405 D01419 D01436 D01440 D01445
D01454 D01471 D01510 D01543 D01549 D01553 D01565 D01571
D01573 D01603 D01606 D01615 D01619 D01634 D01637 D01667
D01673 D01721 D01741 D01748 D01754 D01756 D01794 D01804
D01806 D01810 D01812 D01813 D01822 D01825 D01833 D01849
D01862 D01877 D01886 D01908 D01915 D01943 D01944 D01948
D01966 D01972 D01993 D01998 D02032 D02045 D02061 D02084
D02085 D02110 D02156 D02239 D02276 D02286 D02288 D02338
D02342 D02356 D02363 D02383 D02386 D02460 D02587 D02631
D02705 D02742 D02910 D02939 D02969 D02976 D03115 D03363
D03415 D03450 D03471 D03487 D03492 D03507 D03521 D03664
D03752 D03753 D03765 D03767 D03881 D03892 D03914 D03994
D04004 D04040 D04051 D04079 D04111 D04161 D04195 D04218
D04219 D04221 D04386 D04398 D04399 D04438 D04467 D04488
D04490 D04492 D04532 D04657 D04720 D04733 D04778 D04825
D04897 D04991 D05011 D05077 D05087 D05093 D05107 D05158
D05206 D05442 D05482 D05587 D05606 D05627 D05701 D05711
D05967 D06010 D06102 D06172 D06234 D06329 D06334 D06341
D06392 D06401 D06412 D06536 D06537 D06606 D06622 D06646
D06653 D06665 D07148 D07447 D07450 D07499 D07520 D07526
D07624 D07661 D07796 D07833 D07845 D07858 D07870 D07874
D07892 D07894 D07916 D07946 D07962 D07983 D07992 D08009
D08030 D08031 D08044 D08060 D08074 D08090 D08106 D08146
D08180 D08192 D08194 D08201 D08205 D08215 D08217 D08220
D08225 D08270 D08358 D08365 D08407 D08410 D08411 D08421
D08422 D08435 D08443 D08453 D08482 D08499 D08525 D08600
D08614 D08676 D08677 D08735 D09038

(4) $\mathbb{S}_4^{(i+s)}$: 239 compounds of “Dermatologicals”

D00008 D00054 D00073 D00088 D00104 D00108 D00129 D00133
D00137 D00139 D00140 D00156 D00164 D00165 D00202 D00209
D00233 D00246 D00282 D00292 D00316 D00321 D00322 D00323
D00325 D00328 D00339 D00342 D00351 D00372 D00381 D00385
D00389 D00407 D00418 D00433 D00472 D00494 D00552 D00592
D00689 D00728 D00741 D00770 D00810 D00851 D00858 D00859
D00861 D00862 D00864 D00870 D00882 D00884 D00890 D00975
D00976 D00977 D00979 D00980 D00981 D00982 D00983 D00984
D00985 D01034 D01046 D01047 D01062 D01072 D01073 D01094
D01108 D01112 D01124 D01125 D01132 D01140 D01143 D01242
D01248 D01266 D01272 D01273 D01308 D01327 D01335 D01343
D01361 D01364 D01367 D01384 D01387 D01429 D01458 D01464
D01510 D01516 D01575 D01615 D01619 D01621 D01637 D01664

D01702 D01708 D01711 D01730 D01764 D01768 D01775 D01801
D01820 D01825 D01886 D01905 D01910 D01938 D01948 D01990
D01995 D01998 D02009 D02032 D02053 D02132 D02156 D02184
D02195 D02286 D02287 D02288 D02308 D02375 D02393 D02419
D02435 D02445 D02476 D02477 D02500 D02523 D02524 D02525
D02543 D02554 D02557 D02583 D02589 D02754 D02815 D02845
D02905 D02907 D02923 D03034 D03093 D03101 D03135 D03145
D03290 D03315 D03325 D03383 D03454 D03463 D03473 D03488
D03535 D03538 D03680 D03696 D03697 D03812 D03854 D03884
D03936 D03956 D04201 D04208 D04217 D04218 D04219 D04221
D04281 D04409 D04467 D04624 D04815 D04926 D04934 D05000
D05001 D05002 D05016 D05017 D05072 D05082 D05140 D05225
D05321 D05322 D05407 D05465 D05480 D05529 D05601 D05720
D05815 D06226 D06315 D06527 D06536 D06537 D06543 D06661
D07213 D07495 D07521 D07596 D07668 D07675 D07689 D07753
D07796 D07816 D07822 D07883 D07981 D08183 D08227 D08245
D08313 D08407 D08535 D08543 D08554 D08556 D08645

(5) $\mathbb{S}_5^{(i+s)}$: 157 compounds of "Genito-urinary system and sex hormones"

D00010 D00054 D00066 D00067 D00075 D00079 D00081 D00104
D00111 D00180 D00182 D00185 D00202 D00203 D00220 D00269
D00282 D00289 D00312 D00321 D00327 D00351 D00408 D00419
D00554 D00577 D00581 D00585 D00680 D00682 D00830 D00882
D00884 D00888 D00890 D00898 D00946 D00949 D00951 D00952
D00953 D00957 D00958 D00959 D00970 D00987 D01043 D01046
D01050 D01072 D01073 D01103 D01122 D01139 D01148 D01159
D01217 D01269 D01281 D01294 D01299 D01348 D01352 D01364
D01368 D01374 D01410 D01413 D01462 D01543 D01571 D01575
D01580 D01617 D01639 D01664 D01692 D01829 D01873 D01953
D01965 D01986 D01990 D01995 D02004 D02008 D02073 D02132
D02199 D02256 D02343 D02359 D02367 D02583 D02705 D02725
D03008 D03028 D03062 D03165 D03260 D03399 D03462 D03488
D03538 D03649 D03654 D03820 D03936 D04021 D04061 D04063
D04064 D04065 D04104 D04157 D04317 D04490 D04624 D04672
D04885 D04947 D05016 D05017 D05274 D05321 D05322 D05460
D05679 D06086 D06087 D06425 D06606 D07425 D07456 D07598
D07675 D07726 D07905 D07906 D07961 D08052 D08166 D08167
D08285 D08313 D08346 D08431 D08441 D08465 D08514 D08532
D08560 D08569 D08573 D08574 D08685

(6) $\mathbb{S}_6^{(i+s)}$: 73 compounds of "Systemic hormonal preparations, excluding sex excluding sex hormones and insulins"

D00088 D00089 D00101 D00165 D00249 D00292 D00366 D00385
D00401 D00407 D00472 D00473 D00562 D00930 D00973 D00975
D00976 D00977 D00979 D00980 D00981 D00982 D00983 D00984
D00985 D00986 D01009 D01229 D01510 D01615 D01619 D01637
D01685 D01886 D01948 D01998 D02032 D02105 D02156 D02235
D02286 D02288 D02369 D02983 D03505 D03561 D03594 D03671
D03698 D03699 D04218 D04219 D04302 D04467 D05000 D05001
D05002 D05230 D05729 D06281 D06495 D06672 D06673 D07464
D07616 D07749 D07792 D07796 D07967 D08027 D08241 D08416
D08659

(7) $\mathbb{S}_7^{(i+s)}$: 281 compounds of "Antiinfectives for systemic use"

D00104 D00140 D00144 D00203 D00210 D00211 D00230 D00240
D00258 D00259 D00264 D00273 D00276 D00278 D00285 D00296

D00317 D00322 D00323 D00342 D00344 D00346 D00350 D00351
D00353 D00393 D00398 D00412 D00413 D00423 D00424 D00427
D00433 D00435 D00439 D00445 D00447 D00450 D00578 D00590
D00591 D00592 D00851 D00854 D00855 D00856 D00870 D00872
D00879 D00882 D00894 D00896 D00909 D00911 D00914 D00915
D00916 D00917 D00920 D00925 D00926 D00947 D01047 D01057
D01072 D01073 D01075 D01078 D01079 D01080 D01142 D01144
D01160 D01178 D01195 D01199 D01216 D01235 D01262 D01276
D01283 D01322 D01339 D01361 D01415 D01425 D01426 D01429
D01431 D01501 D01517 D01523 D01526 D01531 D01628 D01629
D01636 D01655 D01680 D01710 D01716 D01739 D01753 D01819
D01863 D01897 D01937 D01954 D01982 D01990 D01995 D02002
D02009 D02119 D02121 D02129 D02132 D02157 D02178 D02184
D02186 D02196 D02199 D02203 D02216 D02222 D02228 D02233
D02267 D02282 D02297 D02299 D02301 D02302 D02305 D02307
D02344 D02345 D02346 D02348 D02352 D02353 D02376 D02406
D02420 D02434 D02435 D02436 D02439 D02450 D02465 D02469
D02471 D02474 D02475 D02496 D02501 D02503 D02505 D02509
D02516 D02517 D02521 D02523 D02524 D02525 D02540 D02541
D02542 D02543 D02545 D02549 D02555 D02861 D02888 D02889
D03039 D03211 D03256 D03262 D03368 D03424 D03428 D03537
D03571 D03640 D03656 D03680 D03706 D03707 D03833 D03837
D03843 D03865 D03895 D04008 D04020 D04049 D04112 D04196
D04254 D04281 D04301 D04859 D04972 D05016 D05017 D05022
D05026 D05045 D05110 D05140 D05271 D05274 D05322 D05351
D05382 D05406 D05407 D05408 D05411 D05460 D05461 D05528
D05949 D05957 D05972 D06057 D06064 D06236 D06298 D06300
D06478 D06558 D06670 D06675 D06676 D07057 D07452 D07486
D07487 D07614 D07635 D07636 D07644 D07645 D07647 D07649
D07650 D07651 D07653 D07654 D07658 D07659 D07675 D07689
D07733 D07782 D07876 D07925 D07995 D08011 D08109 D08120
D08237 D08259 D08268 D08306 D08307 D08380 D08396 D08478
D08483 D08526 D08531 D08533 D08534 D08543 D08557 D08593
D08654

(8) $\mathbb{S}_8^{(i+s)}$: 141 compounds of “Antineoplastic and immunomodulating agents”

D00125 D00155 D00184 D00208 D00214 D00248 D00254 D00266
D00275 D00288 D00341 D00343 D00363 D00369 D00420 D00467
D00468 D00491 D00554 D00567 D00574 D00577 D00583 D00584
D00586 D00749 D00753 D00754 D00946 D00951 D00952 D00960
D00961 D00963 D00964 D00965 D01059 D01061 D01064 D01159
D01161 D01223 D01244 D01270 D01363 D01370 D01441 D01516
D01566 D01747 D01760 D01784 D01790 D01831 D01885 D01907
D01935 D01977 D02020 D02106 D02115 D02131 D02321 D02368
D02494 D02697 D02698 D02714 D02738 D02756 D02815 D02841
D02933 D03033 D03046 D03106 D03150 D03546 D03665 D03786
D03962 D04023 D04024 D04066 D04107 D04187 D04405 D04645
D04687 D04862 D04931 D04988 D05094 D05096 D05333 D05380
D05522 D05589 D05602 D05756 D05807 D05822 D05932 D06066
D06067 D06068 D06117 D06130 D06199 D06247 D06248 D06272
D06304 D06320 D06386 D06397 D06402 D06407 D06414 D06488
D06503 D06619 D06637 D07567 D07671 D07760 D07776 D07866
D07901 D08113 D08166 D08167 D08224 D08423 D08556 D08559
D08603 D08618 D08620 D08675 D08679

(9) $\mathbb{S}_9^{(i+s)}$: 117 compounds of “Musculo-skeletal system”

D00120	D00127	D00132	D00151	D00224	D00241	D00268	D00315
D00402	D00425	D00449	D00452	D00463	D00475	D00492	D00496
D00510	D00567	D00568	D00570	D00755	D00759	D00760	D00763
D00764	D00765	D00767	D00768	D00771	D00813	D00969	D00970
D00991	D00992	D01043	D01049	D01056	D01122	D01183	D01206
D01215	D01252	D01271	D01289	D01305	D01325	D01338	D01344
D01410	D01437	D01475	D01507	D01513	D01545	D01565	D01567
D01581	D01582	D01594	D01671	D01675	D01765	D01767	D01809
D01823	D01824	D01841	D01866	D01975	D02064	D02110	D02207
D02275	D02290	D02292	D02347	D02350	D02355	D02373	D02595
D02709	D02966	D03080	D03254	D03545	D03710	D03714	D03715
D03717	D04102	D04334	D04435	D04486	D04490	D04530	D05078
D05319	D05512	D05513	D05515	D05638	D06073	D06379	D06382
D06388	D06606	D07119	D07281	D07758	D07816	D08104	D08162
D08275	D08484	D08599	D08611	D08614			

(10) $\mathbb{S}_{10}^{(i+s)}$: 382 compounds of "Nervous system"

D00020	D00058	D00059	D00102	D00109	D00130	D00131	D00136
D00217	D00225	D00228	D00252	D00253	D00265	D00280	D00283
D00293	D00308	D00311	D00332	D00338	D00354	D00365	D00370
D00375	D00376	D00387	D00392	D00404	D00415	D00426	D00428
D00454	D00457	D00464	D00470	D00474	D00487	D00495	D00504
D00508	D00524	D00530	D00531	D00532	D00533	D00535	D00536
D00537	D00538	D00539	D00542	D00543	D00544	D00545	D00546
D00547	D00548	D00549	D00552	D00556	D00557	D00560	D00561
D00569	D00604	D00661	D00670	D00675	D00676	D00693	D00698
D00700	D00701	D00704	D00705	D00708	D00709	D00710	D00714
D00741	D00744	D00775	D00781	D00786	D00788	D00789	D00792
D00793	D00795	D00798	D00800	D00801	D00810	D00823	D00838
D00846	D00987	D01000	D01001	D01071	D01096	D01101	D01107
D01110	D01116	D01118	D01150	D01164	D01179	D01190	D01219
D01226	D01228	D01230	D01243	D01245	D01253	D01267	D01268
D01285	D01293	D01295	D01303	D01310	D01314	D01321	D01328
D01351	D01354	D01358	D01371	D01380	D01382	D01391	D01399
D01408	D01412	D01437	D01447	D01451	D01453	D01462	D01465
D01466	D01477	D01485	D01514	D01522	D01546	D01548	D01597
D01630	D01657	D01740	D01744	D01750	D01762	D01772	D01776
D01787	D01793	D01807	D01811	D01871	D01876	D01883	D01898
D01902	D01914	D01973	D02004	D02022	D02071	D02103	D02135
D02162	D02182	D02227	D02236	D02243	D02247	D02252	D02253
D02340	D02357	D02360	D02361	D02408	D02536	D02559	D02561
D02562	D02565	D02566	D02570	D02572	D02573	D02574	D02575
D02578	D02579	D02580	D02604	D02609	D02611	D02612	D02618
D02619	D02621	D02622	D02623	D02624	D02625	D02626	D02627
D02629	D02630	D02643	D02671	D02680	D02683	D02689	D02716
D02717	D02766	D02771	D02780	D02787	D02817	D02824	D02825
D02942	D02991	D02995	D03089	D03102	D03165	D03197	D03199
D03264	D03274	D03385	D03528	D03556	D03562	D03711	D03731
D03783	D03785	D03822	D03825	D03852	D03975	D04034	D04038
D04087	D04088	D04095	D04105	D04127	D04147	D04226	D04257
D04264	D04292	D04314	D04605	D04650	D04741	D04747	D04749
D04750	D04765	D04882	D04924	D04965	D04985	D04999	D05028
D05040	D05113	D05133	D05156	D05181	D05200	D05290	D05339
D05340	D05375	D05462	D05478	D05523	D05575	D05593	D05621
D05623	D05626	D05740	D05768	D05775	D05928	D05938	D06007
D06147	D06168	D06282	D06327	D06339	D06367	D06536	D06537
D06573	D06623	D07122	D07132	D07286	D07339	D07349	D07441

D07445	D07448	D07511	D07591	D07593	D07678	D07704	D07727
D07791	D07809	D07837	D07875	D07881	D07906	D07977	D07984
D07993	D08047	D08054	D08070	D08098	D08100	D08116	D08133
D08170	D08174	D08181	D08187	D08195	D08226	D08233	D08246
D08255	D08257	D08261	D08283	D08288	D08339	D08343	D08349
D08422	D08425	D08430	D08447	D08456	D08473	D08489	D08490
D08549	D08555	D08575	D08588	D08623	D08625	D08626	D08634
D08636	D08638	D08670	D08687	D08690	D09569		

(11) $\mathbb{S}_{11}^{(i+s)}$: 75 compounds of "Antiparasitic products, insecticides and repellents"

D00131	D00134	D00236	D00360	D00368	D00372	D00436	D00460
D00471	D00486	D00488	D00489	D00534	D00582	D00804	D00806
D00807	D00808	D00828	D00832	D00833	D00834	D00862	D01138
D01393	D01426	D02127	D02145	D02379	D02387	D02448	D02472
D02480	D02481	D02482	D02483	D02486	D02489	D02922	D03028
D03135	D03469	D03538	D03623	D03985	D04140	D04200	D04895
D05016	D05017	D05170	D05274	D05443	D06051	D06114	D06224
D06238	D06239	D07106	D07367	D07530	D07761	D07763	D07785
D07825	D07883	D08033	D08050	D08114	D08179	D08357	D08420
D08428	D08451	D08460					

(12) $\mathbb{S}_{12}^{(i+s)}$: 190 compounds of "Respiratory system"

D00074	D00164	D00195	D00221	D00227	D00234	D00242	D00246
D00292	D00324	D00337	D00364	D00385	D00411	D00472	D00494
D00521	D00552	D00687	D00689	D00691	D00858	D00975	D00980
D00981	D00982	D00983	D00984	D00985	D01016	D01021	D01062
D01106	D01117	D01123	D01140	D01143	D01172	D01174	D01242
D01260	D01288	D01324	D01332	D01343	D01347	D01360	D01385
D01389	D01427	D01430	D01459	D01475	D01478	D01479	D01490
D01506	D01510	D01575	D01576	D01608	D01615	D01621	D01627
D01635	D01637	D01703	D01708	D01713	D01717	D01771	D01773
D01778	D01782	D01786	D01801	D01828	D01872	D01929	D01946
D01948	D01957	D01998	D02017	D02023	D02032	D02101	D02151
D02156	D02172	D02182	D02195	D02212	D02230	D02286	D02290
D02327	D02354	D02396	D02419	D02589	D02610	D02732	D02760
D02822	D02884	D02950	D03051	D03166	D03184	D03290	D03325
D03402	D03454	D03463	D03535	D03557	D03580	D03585	D03622
D03693	D03742	D03854	D03888	D03898	D04080	D04089	D04157
D04163	D04441	D04980	D05006	D05037	D05130	D05140	D05321
D05366	D05429	D05484	D05718	D05730	D05744	D05792	D06103
D06104	D06107	D06171	D06315	D06393	D06536	D06537	D06543
D07125	D07409	D07482	D07483	D07495	D07534	D07543	D07547
D07617	D07662	D07668	D07753	D07765	D07796	D07803	D07881
D07929	D07958	D07981	D07990	D08045	D08090	D08163	D08183
D08227	D08229	D08293	D08300	D08322	D08353	D08365	D08368
D08387	D08449	D08570	D08578	D08645	D08684		

(13) $\mathbb{S}_{13}^{(i+s)}$: 202 compounds of "Sensory organs"

D00008	D00010	D00043	D00054	D00088	D00104	D00127	D00133
D00140	D00164	D00165	D00184	D00210	D00221	D00292	D00317
D00342	D00356	D00385	D00391	D00397	D00433	D00450	D00472
D00518	D00524	D00604	D00652	D00655	D00667	D00728	D00741
D00810	D00813	D00851	D00858	D00862	D00870	D00882	D00884
D00955	D00956	D00973	D00975	D00976	D00977	D00980	D00981
D00982	D00983	D00984	D00985	D00999	D01004	D01016	D01021
D01037	D01072	D01089	D01162	D01196	D01261	D01273	D01332

D01361 D01367 D01451 D01452 D01475 D01510 D01512 D01565
D01578 D01594 D01615 D01619 D01621 D01637 D01689 D01713
D01717 D01724 D01730 D01733 D01768 D01820 D01821 D01825
D01886 D01948 D01954 D01964 D01976 D01995 D01998 D02009
D02024 D02032 D02070 D02071 D02110 D02119 D02133 D02156
D02157 D02182 D02184 D02193 D02216 D02286 D02288 D02289
D02290 D02349 D02374 D02393 D02418 D02523 D02524 D02525
D02541 D02542 D02543 D02549 D02589 D02706 D02724 D02750
D02842 D03011 D03163 D03243 D03262 D03463 D03538 D03648
D03696 D03814 D03826 D03947 D04221 D04244 D04281 D04301
D04467 D04488 D04760 D04762 D04934 D05130 D05140 D05143
D05319 D05322 D05408 D05478 D05512 D05513 D05729 D05763
D05872 D05947 D05957 D06274 D06298 D06300 D06543 D07148
D07483 D07486 D07526 D07540 D07624 D07668 D07675 D07689
D07749 D07753 D07759 D07796 D07816 D07840 D07871 D07890
D07929 D08011 D08030 D08104 D08115 D08120 D08237 D08250
D08261 D08268 D08293 D08322 D08365 D08422 D08448 D08578
D08600 D08684

(14) $\mathbb{S}_{14}^{(i+s)}$: 128 compounds of “Various”

D00003 D00004 D00014 D00083 D00096 D00114 D00167 D00221
D00226 D00294 D00509 D00517 D00697 D00707 D01014 D01016
D01027 D01060 D01091 D01099 D01108 D01137 D01181 D01187
D01200 D01311 D01312 D01313 D01346 D01376 D01388 D01421
D01459 D01474 D01555 D01563 D01570 D01631 D01644 D01645
D01646 D01707 D01714 D01719 D01755 D01797 D01817 D01843
D01880 D01884 D01893 D01936 D01940 D01979 D01983 D01999
D02007 D02015 D02025 D02029 D02036 D02053 D02108 D02161
D02284 D02312 D02402 D02409 D02418 D02457 D03355 D03443
D03669 D03670 D03730 D03770 D03826 D03902 D03947 D04220
D04283 D04284 D04288 D04291 D04357 D04420 D04560 D04562
D04563 D04567 D04570 D04572 D04573 D04577 D04582 D04586
D04596 D04598 D04599 D04604 D04656 D04667 D04929 D05019
D05215 D05436 D05439 D05590 D05647 D05795 D05845 D05860
D05865 D05870 D05940 D05962 D06027 D06029 D06030 D06039
D06042 D06049 D06177 D06339 D08027 D08247 D08249 D08548
